# Solution and Solid-State Photophysical Properties of Positional Isomeric Acrylonitrile Derivatives with Core Pyridine and Phenyl Moieties: Experimental and DFT Studies

**DOI:** 10.3390/molecules26061500

**Published:** 2021-03-10

**Authors:** Armando Castillo, Paulina Ceballos, Pilar Santos, Margarita Cerón, Perumal Venkatesan, Enrique Pérez-Gutiérrez, Martha Sosa-Rivadeneyra, Subbiah Thamotharan, Maxime A. Siegler, María Judith Percino

**Affiliations:** 1 Unidad de Polímeros y Electrónica Orgánica, Instituto de Ciencias, Benemérita Universidad Autónoma de Puebla, Val3-Ecocampus Valsequillo, Independencia O2 Sur 50, San Pedro Zacachimalpa 72960, Mexico; armando.castillo@alumno.buap.mx (A.C.); paulina.ceballos@correo.buap.mx (P.C.); pilar.santos@alumno.buap.mx (P.S.); margarita.ceron@correo.buap.mx (M.C.); venkatesan.perumal@correo.buap.mx (P.V.); enrique.pgutierrez@correo.buap.mx (E.P.-G.); 2 Facultad de Ciencias Químicas, Benemérita Universidad Autónoma de Puebla (BUAP), 14 Sur Esquina San Claudio, San Manuel, Puebla 72570, Mexico; martha.sosa@correo.buap.mx; 3 Biomolecular Crystallography Laboratory, School of Chemical & Biotechnology, Department of Bioinformatics, SASTRA Deemed University, Thanjavur 613401, India; thamu@scbt.sastra.ac.in; 4 Department of Chemistry, Johns Hopkins University, New Chemistry Building, 3400 N. Charles St., Baltimore, MD 21218, USA; msiegle2@jhu.edu

**Keywords:** optical properties, solution and solid state, acrylonitrile derivatives, DFT studies, PIXEL, MEPS, NBO

## Abstract

The compounds **I** (*Z*)-2-(phenyl)-3-(2,4,5-trimethoxyphenyl)acrylonitrile with one side (2,4,5-MeO-), one symmetrical (2*Z*,2′*Z*)-2,2′-(1,4-phenylene)bis(3-(2,4,5-trimethoxyphenyl)acrylonitrile), **II** (both sides with (2,4,5-MeO-), and three positional isomers with pyridine (*Z*)-2-(pyridin-2- 3, or 4-yl)-3-(2,4,5-trimethoxyphenyl)acrylonitrile, **III**–**V** were synthetized and characterized by UV-Vis, fluorescence, IR, H^1^-NMR, and EI mass spectrometry as well as single crystal X-ray diffraction (SCXRD). The optical properties were strongly influenced by the solvent (hyperchromic and hypochromic shift), which were compared with the solid state. According to the solvatochromism theory, the excited-state (*μ_e_*) and ground-state (*μ_g_*) dipole moments were calculated based on the variation of Stokes shift with the solvent’s relative permittivity, refractive index, and polarity parameters. SCXRD analyses revealed that the compounds **I** and **II** crystallized in the monoclinic system with the space group, *P*2_1_/*n* and *P*2_1_/*c*, respectively, and with *Z* = 4 and 2. **III**, **IV**, and **V** crystallized in space groups: orthorhombic, *Pbca*; triclinic, *P*-1; and monoclinic, *P*2_1_ with *Z* = 1, 2, and 2, respectively. The intermolecular interactions for compounds **I**–**V** were investigated using the CCDC Mercury software and their energies were quantified using PIXEL. The density of states (DOS), molecular electrostatic potential surfaces (MEPS), and natural bond orbitals (NBO) of the compounds were determined to evaluate the photophysical properties.

## 1. Introduction

Solid state materials whose optical properties are susceptible to changes by external stimuli (mechanical forces, solvent, acid vapors, heat and light) are of ongoing interest because they are used in optical information storage, fluorescent switches, memory devices, and optoelectronic devices [1,2]. Understanding and being able to predict the photophysical and electronic properties of a material is essential for its application [3,4,5]. In general, the properties of individual molecules can be predicted with confidence, yet their behaviors as aggregates, crystals, or in solution remains poorly understood for applications in many areas of science and technology. Therefore, the molecular arrangement and the intermolecular interactions in the solid state, as well as with the solvent in solution, are important parameters to correlate with their optical properties [6,7]. In some cases, the solvent causes the fluorescence intensity to decrease (known as quenching), which occurs when the fluorophore in the excited state is deactivated when it meets a molecule of the solvent [8]. Another effect is that the polarity of a solvent changes the emission wavelength of a fluorophore leading to the effect known as solvatochromism. Although the solvent plays an important role in many areas of synthetic chemistry, molecular biology, and materials design, the effect of the solvent in many chemical reactions and in structural characterizations has been given little attention [9,10,11,12,13]. The solvent effects on optical properties rely on solvent properties, such as dipole moment, electronic polarizability, hydrogen bond (HB) donor and acceptor capability, intermolecular electrostatic interactions, etc. [14]. On the other hand, the optical properties of the materials may be related to intermolecular or intramolecular π complexing interactions, in which aromatic rings participate, at the supramolecular level [14,15,16,17,18,19,20]. These types of interactions are known as non-covalent (NCI), and are due to π stacking and to X-H/π interactions (X = C, O, N, S). These interactions are suitable for charge transfer of neutral molecules and donors rich in π electrons and various acceptors deficient in π-electrons. The stacking structure or non-stacking structure is stabilized by a π bond or other weak forces and plays an important role in the supramolecular system [21,22,23,24,25]. Noncovalent interactions are crucial for efficient performance of optoelectronic devices such as organic light emitting diodes and organic thin film transistors [26,27,28,29,30]. Aromatic stacking is important to control solvent-dependent nucleation in crystal growth of the material [31]. Therefore, in solution the solute-solvent and solvent-solvent interactions affect the π interactions of aromatic rings [32]. The role of the effect of solvents on the absorption and fluorescence of organic compounds has been extensively studied [33,34,35,36,37,38,39,40,41]. Spectral shifts to either the blue or the red as well as in the intensity of the emission can be due to effects of solvents polarity [7,9], the variations in temperature [33,42], and the pH values [34,35,36].

The optical properties can also be altered by substituents located in various positions around the molecular structure [41,43,44]. Especially, the optical properties of compounds containing a pyridine ring and with α-cyanostilbenes with dimethylamine, diphenylamine, halogen atoms and *N*-ethylcarbazole substituents have been reported [45,46,47,48,49,50,51,52,53]. It has been established that molecules are not emitters in solution (SCQ) [54,55], which prompted us to question why are such compounds not emitters? We proposed a study of a compound in both the solution and the solid states with evaluation of the physicochemical properties of solvents on the properties of luminophores. In the solid state, the effect of the intermolecular interaction energy could be correlated with the optical properties. 

**Figure 1:**
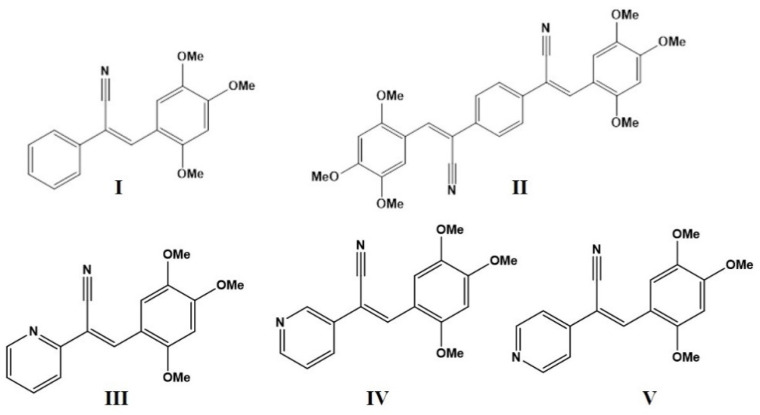
Chemical structures of compounds **I**–**V.**

In the present work, we carry out the synthesis, characterization, and investigation in solution to estimate fundamental and excited dipole moments. We have also examined the solid-state optical properties of five compounds: **I,** (*Z*)-2-(phenyl)-3-(2,4,5-trimethoxyphenyl)acrylonitrile; **II**, (2*Z*,2′*Z*)-2,2′-(1,4-phenylene)bis(3-(2,4,5-trimethoxy-phenyl)acrylonitrile); **III,** (*Z*)-2-(pyridin-2-yl)-3-(2,4,5-trimethoxyphenyl) acrylonitrile; **IV**, (*Z*)-2-(pyridin-3-yl)-3-(2,4,5-trimethoxyphenyl)acrylonitrile; and **V**, (*Z*)-2-(pyridin-4-yl)-3-(2,4,5-trimethoxyphenyl)acrylonitrile (Figure 1). The compounds were designed with one being symmetrical (both sides with (2,4,5-MeO-), another with only one side (2,4,5-MeO-), and three positional isomers with pyridine, to evaluate the effects of *ortho*, *meta,* and *para* located substituents. The intermolecular interaction was examined using the CCDC [56] Mercury software and their energies were determined using PIXEL [57,58,59]. We performed density of states (DOS), molecular electrostatic potential surfaces (MEPS), and natural bond orbital (NBO) analyses of the compounds to evaluate the effect of the substituents on the optical properties as well as the effects of intermolecular and other interactions on the charge transfer.

## 2. Result and Discussion

### 2.1. Synthesis

The synthetic methodology for **I** (*Z*)-2-(phenyl)-3-(2,4,5-trimethoxyphenyl)acrylonitrile, **II** (2*Z*,2′*Z*)-2,2′-(1,4-phenylene)bis(3-(2,4,5-trimethoxyphenyl)acrylonitrile), **III** (*Z*)-2-(pyridin-2-yl)-3-(2,4,5-trimethoxyphenyl)acrylonitrile, **IV** (*Z*)-2-(pyridin-3-yl)-3-(2,4,5-trimethoxyphenyl)acrylonitrile, and **V** (*Z*)-2-(pyridin-4-yl)-3-(2,4,5-trimethoxyphenyl)acrylo-nitrile compounds (Figure 1), followed the procedures described in the ESI. Their characterizations by ^1^H-NMR, electronic impact spectrometry, solubility, and crystallization conditions for SCXRD characterization are included in the ESI.

The reaction conditions used for the compounds, including catalyst, stoichiometric ratio, and temperature, were the same (Table S1). The reaction time was shorter for compound **II**, whereas compounds **I**, **III**, **V** required the same reaction time. Only for compound **IV** was the time longer, which is an indication of the reactivity of pyridine in the *meta* position. The yields were ranked in the order **II** > **I** > **V** > **III** > **IV**. The crystallization conditions and the whole characterization by IR, ^1^H NMR, and EI are shown in Figures S1–S3 and Tables S2–S4 (ESI).

### 2.2. Absorption Properties of **I**–**V** in Solution and Solid State

#### 2.2.1. The Effect of the Solvent on the Absorption Spectra

The UV/Vis absorption spectra were recorded in various solvents (non-polar, polar aprotic and polar protic) [53], at the same concentration and at room temperature (Figures S4–S8). The solvents used were DMSO (**1**); methanol (MeOH, **2**); acetonitrile (AcCN, **3**); acetone (**4**); THF (**5**); ethyl acetate (EtOAc, **6**); CHCl_3_(**7**); and a 70:30 MeOH:acetone mixture (**8**) (chosen because acetone and methanol affect the absorption of the compounds).

**Figure 2:**
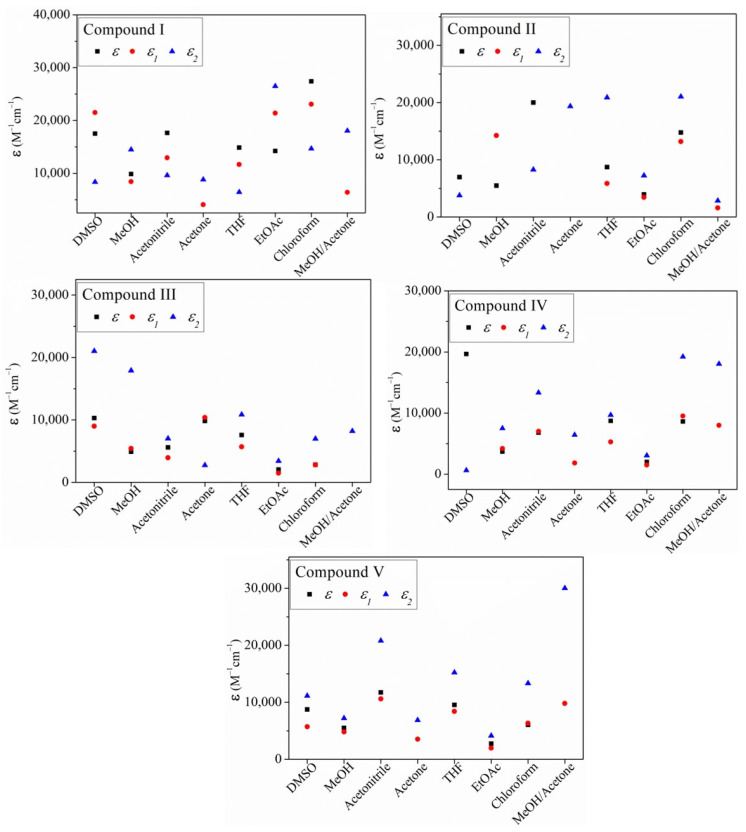
Molar extinction coefficient of the compounds **I** and **II**, **III**, **IV**, **V** and in solvents (**1**) DMSO, (**2**) MeOH, (**3**) AcCN, (**4**) acetone, (**5**) THF, (**6**) EtOAc, (**7**) CHCl_3_, and (**8**) MeOH:acetone (70:30). Concentration of 0.001 mM.

The spectra of the compounds in most of the solvents (Figures S4–S7) showed three absorbance peaks with different intensities: one band (peak) in the range of 243–258 nm with molar coefficient **ε**, the second one (peak) was in 305–316 nm (**ε_1_**), and the third peak in the range of 385–398 nm (**ε_2_**) and in the range of 413–415 nm. These peaks are typically assigned to n→π* and π→π* electronic transitions, but the solvent effect on absorption properties was greater on the absorption maxima intensity (hyperchromic and hypochromic shift) than on absorption wavelength for the **I**–**V** compounds, and only in some cases a very small wavelength shift was observed. We plotted the molar extinction coefficients (**ε, ε_1_, ε_2_**) against solvent for **I**–**V** (Figure 2) and the (**ε, ε_1_, ε_2_**) values are summarized in Table S5.

For **I,** the **ε_2_** decreased according to the solvent dielectric constant (Table S7), with the behaviors of **ε, ε_1_** and **ε_2_** being ranked **1** > **2** > **3** > **4** and > **5**. But **ε_2_** increased to 27,000 M^−1^cm^−1^ in **6** and showed a slight decrement to (15,000 M^−1^cm^−1^) in solvents **7** and **8**. But **ε** is zero in solvents **4** and **8**. The compound **II** has a more rigid molecular structure (Figure 1), and with extension of the large conjugated system, unlike compound **I**. The **ε, ε_1_** and **ε_2_** molar coefficient extinctions and absorption spectra are shown in Figure 1 and Figure S5, respectively, and the values are summarized in Table S5. The absorbance wavelength underwent a bathochromic effect, due to the conjugated system extension. But unlike **I**, the compound **II ε_2_** was lower in solvents **1**, **2** and **8** (hypochromic behavior <5000 M^−1^cm^−1^) and began to rise as the solvent dielectric constant decreased in the solvents **3**, **4**, **5** and **7.** In solvent **2** (polar protic solvent), the wavelength of maximum absorption was at 341 nm (blue shift). The value of **ε** was lower in most of the solvents save in **3** and **7**, with the value being between 15,000–20,007 M^−1^ cm^−1^. Whereas the absorption band at 416 nm tends to increase its intensity (hyperchromic effect) in polar and non-polar media such as **4**, **5** and **7**, **ε_2_** ≈ 20,007 M^−1^cm^−1^.

For compound **III**, the **ε**, **ε_1_**, **ε_2_** decreased according to the decrease in the dielectric constant of the solvent, as noted for compound **I**, but the values did not increase in nonpolar solvents (**5**, **6**, **7**) unlike **IV**. Also, the **ε_2_** of the **III** isomer (*ortho* position) was highest in solvent **1** (hypochromic effect) and the lowest intensity (**ε, ε_1_,ε_2_**) < 5000 M^−1^cm^−1^. In some solvents, non-value for **ε** is reported because for the used concentration (0.001 mM), the absorption spectra are almost zero. Depending on the used solvent, also hypochromic effect was observed, and in solvents **3** and **4**, the absorption wavelength suffered a small blue shift (Figure S6). For the compound **IV** isomer with the adduct in the *meta* position, **ε_1_** and **ε_2_** values in solvent **1** were approximately zero, but **ε** was not (Figure 1 and see also Figure S7 which depicts absorption spectra). **ε_2_** begins to increase in solvents **2**, **3** and it remains at lower values in solvents **4**, **5**, **6,** with the highest values observed with **7** and **8**. This outcome is an indication of the hypochromic effect observed in polar protic, aprotic, and nonpolar solvents on **ε_2_** (13,000−5000 M^−1^cm^−1^). For compound **V**, the most intense band appeared almost at the same wavelength (395–397 nm), but with a slight red shift due to the *para* position of the nitrogen of the pyridine. The **ε, ε_1_** and **ε_2_** molar coefficients decreased in most of the solvents. The **ε** and **ε_1_** value were between 13,000–7500 M^−1^cm^−1^ in **1**, **2**, **4**, **6**, **7**, and the **ε, ε_1_** and **ε_2_** were closer to zero in **4** and **6** (EtOAc). The **III**–**V** with **I**–**II** outcomes may be attributed to the electron pair of the nitrogen atom and the fact that the solvents **4** and **6** completely affected the absorption unlike in **I**, which was more affected by solvent **4**. Compound **II** was not affected in solvents **4** and **6** while **ε, ε_1_** were affected. The results show that the nitrogen free electron pair of the pyridine group causes the changes in the absorbance intensity [60,61,62,63]. The observed hyperchromic and hypochromic changes were caused by the interaction with solvents, but particularly solvents with oxygen atoms, which can act as auxochromes, that is, as electron donators (**EDG**). Because the phenyl group does not interact strongly with solvents, it is possible that in solution there is a free rotation of the phenyl group, and this affects the absorption peak between 366–381 nm (Figure S4). Free rotation is restricted in compound **II**, so it is possible that free rotation as a function of solvent causes the observed hypochromic and hyperchromic effects, while the bathochromic shift is also the result of the presence of the extended conjugation in the molecule. The change in the position of the absorption maxima is due to conjugation [38].

#### 2.2.2. Absorption Properties by Theoretical Calculation

**Figure 3:**
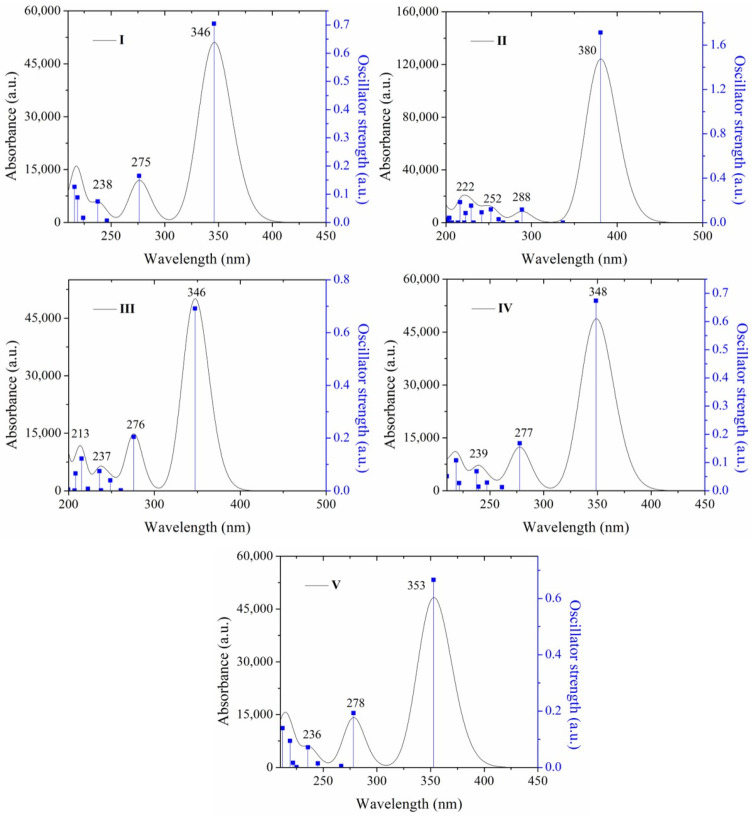
Absorption spectra of **I**–**V** calculated at a theoretical level of M062x/cc-pVTZ in the gas state.

**Figure 4:**
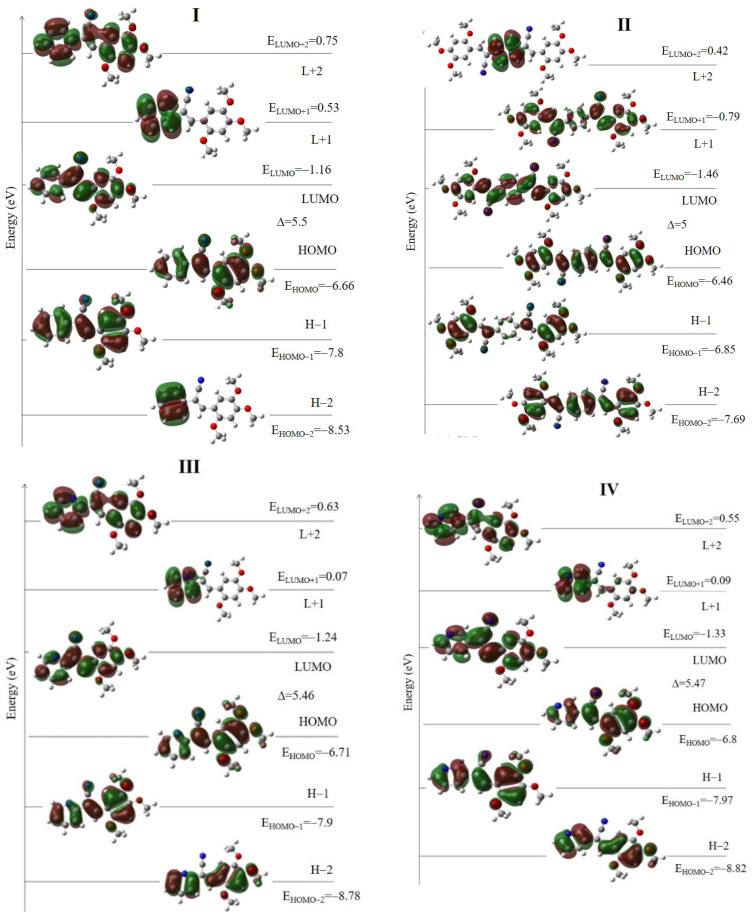

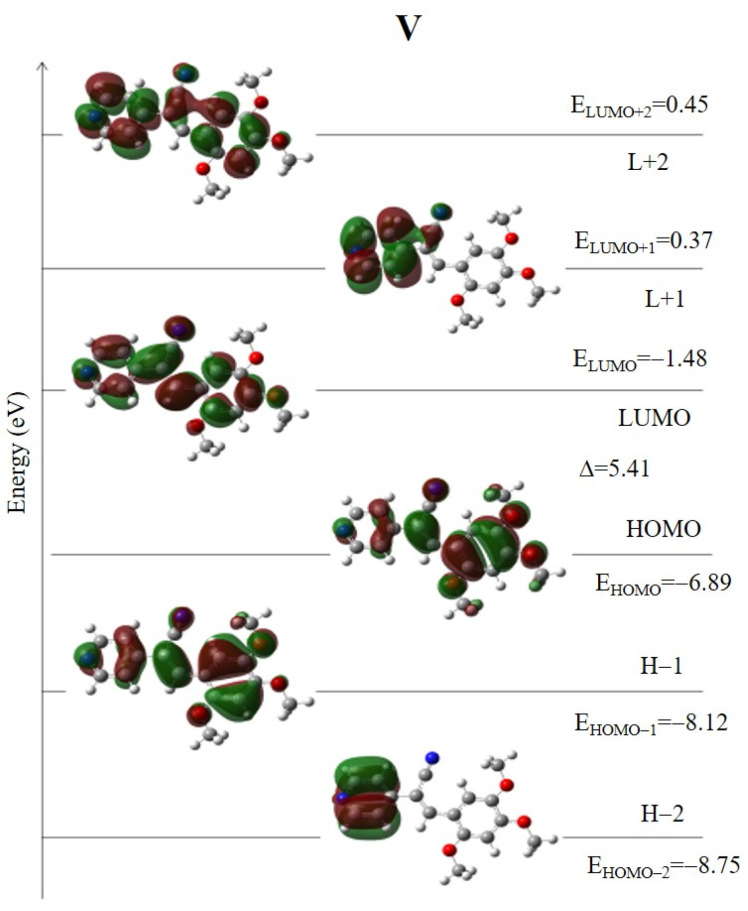
Frontier molecular orbital diagrams of **I**–**V** compounds.

**Figure 5:**
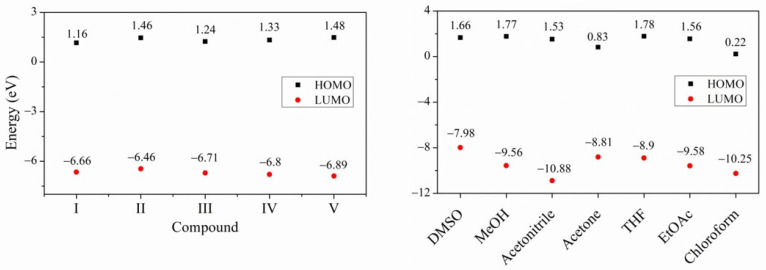
Schematic diagrams of HOMO and LUMO DFT predicted values of compounds **I**–**V** and solvents used in the study. DMSO, MeOH, AcCN, acetone, THF, EtOAc, CHCl_3_.

The density functional theory (DFT) calculations were conducted at the M06-2X-/cc-pVTZ level [63]. The ground state electronic structures and geometries of the **I**, **II** and the positional isomers **III**–**V** were calculated. The DFT optimized structures by Gaussian 09 program [64] of **I**–**V** are in good accordance with the crystal molecular structures, which is described in Section 3. To understand the electronic transition of **I**–**V** compounds, the theoretical UV-Vis spectra were calculated in the gas phase (Figure 3) and in different solvents (**1**–**8**) (Figures S9–S13). The computed electronic values such as absorption wavelength (λ), excitation energy (*E*), oscillator strengths (*f*) are shown in Table 1. The calculated UV-vis spectra for **I**–**V** compounds showed the transitions for the three absorption maxima detected (Figures S4–S8). The λ absorption for **I** at 387 nm is attributed mainly to a HOMO→LUMO transition, but **II** the absorption at 416 nm is due to HOMO − 1→LUMO + 1 and HOMO→LUMO, which is an indication of intra-molecular charge transport (ICT). Also, the data indicate the different MO involved for the absorptions observed in the range of 220–300 nm. **I** and **II** frontier molecular orbitals (FMO) for absorption in the range 290–324 nm are due to HOMO − 1→LUMO, and H − 3→L + 1, H − 2→LUMO H − 1→L + 1 respectively, whereas in the range of 235–278 nm there is a greater number of orbitals involved in the transition, for **I** with the higher oscillator value are H − 3→LUMO, HOMO→L + 1(17) HOMO→L + 2, HOMO→L + 3, and for **II** HOMO→L + 3, HOMO→L + 5 HOMO→L + 3, HOMO→L + 5 (Table 1). For the isomers in -*ortho* **III**, -*meta* **IV** and -*para* **V**, the respective λ_abs_ at 393 nm, at 387 nm, and at 393 nm are attributed mainly to a HOMO→LUMO transition and the absorption around 300 nm to HOMO − 1→ LUMO. The calculated FMO’s are shown in Figure 4. The transitions around of 243–270 nm are different for each isomer in the FMO. The HOMO − 2 electron density is different, which is due to the position of the nitrogen (Figure 4). For **I**, the HOMO electronic density is localized in the atoms of 2,4,5-TMeO-phenyl ring and on the acrylonitrile group, whereas that the LUMO the electronic density is mainly localized at the phenyl moiety. But in **II** the HOMO electronic density involved the atoms of the double bonds, indicating the conjugation along the whole structure, but in LUMO is concentrated on the central phenyl ring and encompasses the acrylonitrile moiety, forming a quinoid structure. Thus, the electron densities in the **I**–**V** structures are different, and therefore the interaction with the solvent must also be different, regardless of the medium polarity, and therefore the optical properties of **I**–**V** are affected. Concerning the frontier molecular orbital theory, energy values are related to chemical reactivity and electronic transition [65,66]. The energy of the highest occupied molecular orbital (E_HOMO_) measures the tendency towards the donation of an electron by a molecule. Therefore, higher values of E_HOMO_ indicate a better tendency towards the donation of electron and energy of the lowest unoccupied molecular orbital (E_LUMO_) indicates the ability of the molecule to accept electrons. Comparing the FMO energy values of the acrylonitrile derivatives, **I**–**V** containing 2,4,5-TMeO- with reported compounds [46,47,48,49,50,67] substituted with F, Cl, Br, -N(CH3)_2_, -N(Ph)_2_, -Cz, chalcones, and ring phenyl ring or pyridine ring in *ortho*, *meta* and *para* positions, we found that E_LUMO_ values indicated **I**–**V** are better electron acceptors than electron donors (Figure 5 and Figure S14) and are able to interact with solvents because the HOMO and LUMO values are very close. The experimental and calculated absorption wavelengths in all solvents are summarized in Table S6 and the calculated absorptions in Figures S11–S13, as well as the HOMO and LUMO of the solvents, Figure S15, and Table S7.

The E_LUMO_ is higher than the compounds with -N(CH_3_)_2,_ -Cz, and -N(Ph)_2_, (Figure S14). The **I**–**V** FMOs energy values are close to compounds substituted with halogens atoms and to three chalcone derivatives with the MeO-group [67]. We can derive additional information, the HOMO and LUMO energy gap, which describes the chemical softness-hardness of a molecule [61]. The molecules having a small energy gap are known as soft and those having a large energy gap are known as hard molecules. The hard molecules are not more polarizable than the soft ones because they need great energy to achieve excitation [68]. The hardness value (*η*) is calculated using the following equation [69]:

(1) $$\eta =\frac{-E_{HOMO}+E_{LUMO}}{2}$$

The *η* values (Table 2) indicated that **I**–**V** could be considered as hard molecules compared with molecules substituted with -N(CH_3_)_2_, -N(Ph)_2_, -Cz, chalcones. 

The dipole moment is also an important parameter (Table 3). The FMO of the solvents **1**–**7** used is also shown in Figure S15, as well as the energy gap data (Table S10), which is an indication that the solvents are harder than the compounds **I**–**V** (Figure 5). This factor can disturb the charge density of the molecules and the effect on the absorbance of the compounds in the presence of protic, non-protic, and non-polar solvents such as THF and EtOAc. 

**Figure 6:**
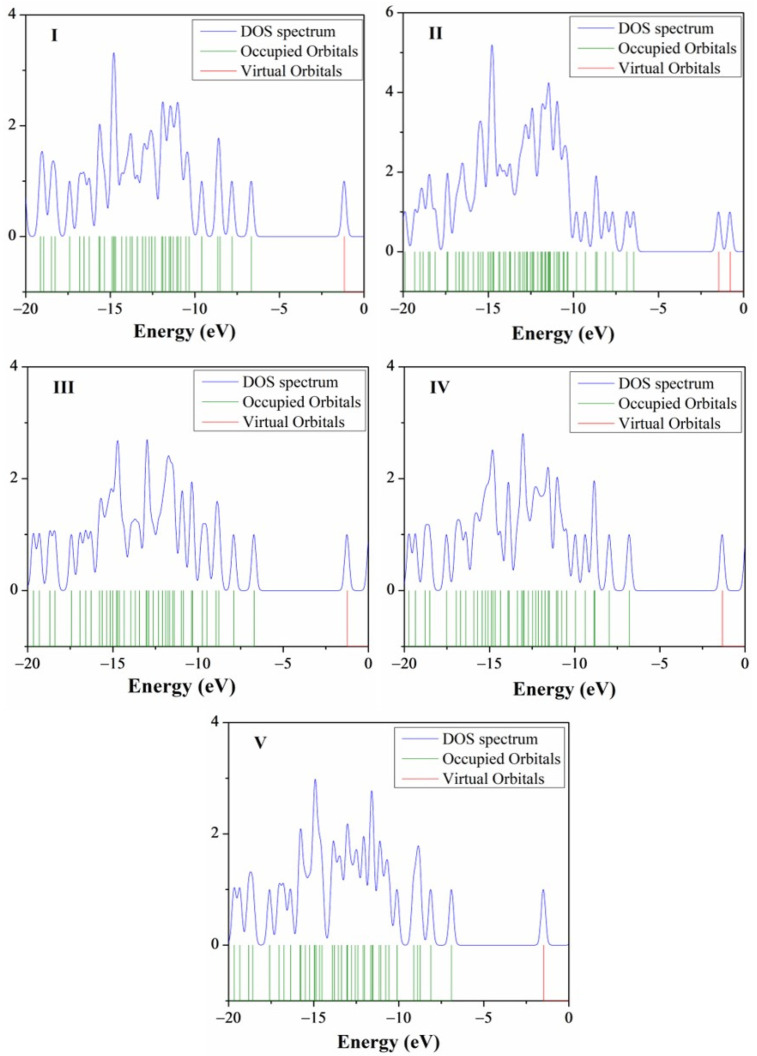
Density of states (DOS) diagrams of **I**–**V** compounds.

As shown in the density of states spectra for each molecule (Figure 6), the results support the energy gap calculated by HOMO-LUMO analysis [64]. The green and red lines in Figure 6 indicate the HOMO and LUMO levels, respectively. A comparison of **I** DOS with the **II** DOS spectrum indicates that the states for **II** are split. The absorption band for the **II** related to the So→S_1_ transition is bathochromically shifted with respect to **I**. This outcome could be attributed to **II** undergoing transitions to lower energy compared to the transitions of **I** that shift to the blue. The DOS spectra of the **III** and **IV** are similar except **V** shows DOS that are occupied at energy (−10 to −5 eV. Figure 6).

#### 2.2.3. Absorption Spectra in the Solid State

**Figure 7:**
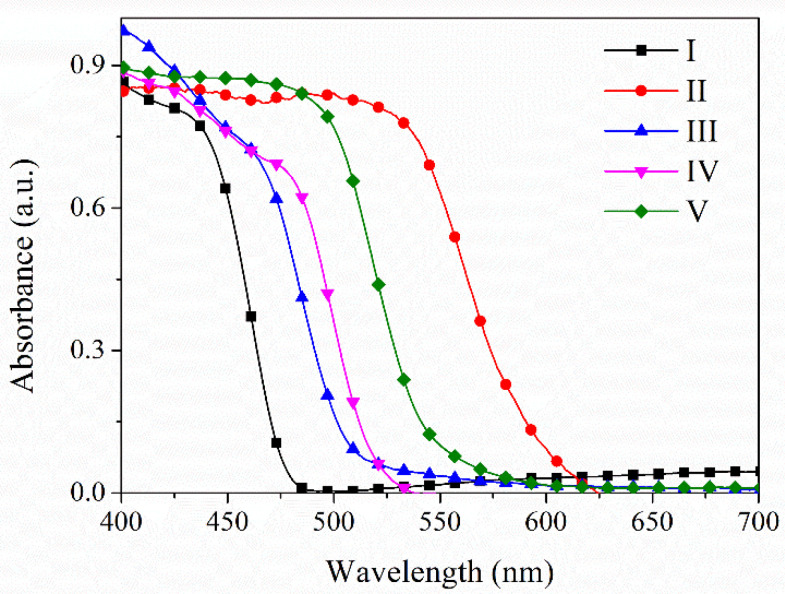
Absorbance spectra of compounds **I**–**V** in solid state at room temperature.

The absorption maxima exhibited a red shift in the solid state compared to absorbance maxima observed in solution (Figure 7). The shift in absorption wavelength of **I** at 436 nm is of 100 nm compared with **II** at 534 nm, which could be correlated to the intermolecular interactions in the solid state. Between the isomers **III**–**IV** the difference is only 30–40 nm (Table S10).

#### 2.2.4. Emission Spectra in Solution and Solid State

**Figure 8:**
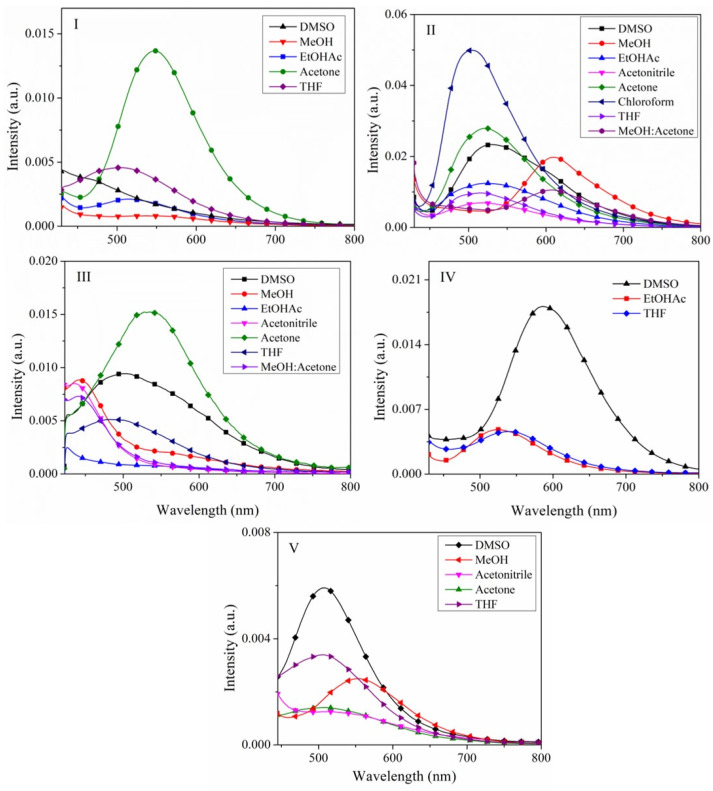
Emission spectra of compound **I**–**V** in solvents DMSO, MeOH, AcCN, acetone, THF, EtOAc, CHCl_3_, and MeOH:acetone (70:30). Concentration of 0.001 mM.

The solvent effect study on the absorption and emission spectra was performed because the compounds **I, III**–**V** and the positional isomers showed a low intensity emission in different solvents (solvent caused quenching (SCQ), images of the solution’s emission under UV lamp, Figure S11), i.e., in some solvents no emission was detected. The fluorescence spectra of the **I**–**V** solutions are shown in Figure 8. Table 2 summarizes the Stokes shift for all compounds, dipole moment by DFT calculations (Figure S12), and quantum yield. Usually, it has been reported that the sensitivity of the Stokes shift to solvent polarity is the reason why fluorescence emission spectra are frequently used to estimate the polarity of the environment surrounding the fluorophore [71]. Solvent-caused quenching (SCQ) of **I** was observed in MeOH, AcCN, CHCl_3_ and (MeOH/acetone). The maximum intensity of emission was in acetone and THF. Unlike **II**, the emission intensity maximum of **I** was at 468–513 nm (Figure 8 and Table S8), and although the dipole moment is approximately zero, SCQ was not observed. Between isomers of approximately the same dipole moment (Table 3), the effect of the solvent on fluorescence differed (Figure 8). SCQ was observed in **III** in **6** and **7**, **IV** in DMSO, MeOH, EtOAc and MeOH/acetone solvents, and **V** in EtOAc, CHCl_3_ and MeOH/acetone. All emission wavelengths for **I**–**V** are summarized in Table S8. The solvent effect on a fluorophore could be described by plotting of the Stokes Shift (ῡa − ῡ_f_) (Table S8) versus the orientation palatability F (ε, n) (Table S9) according to the Lipper Equations (2) and (3), where (ε) represents the dielectric constant and (*n*) the refractive index of the solvents [71]. In the present study, we found no single, typical behavior, which is an indication of several types of interactions including hydrogen bonding, charge shift, solvent polarity, conformational changes, etc. All these interactions can result in spectral shifts and can indicate the effects of the environment on the energy of the excited state. Furthermore, **I**–**IV** can be fluorescent or nonfluorescent in different states. 

Lippert’s equation:

(2) $$\bar{\upsilon }a-\bar{\upsilon }f=\mathrm{SF}\;\left(\varepsilon ,\;n\right)+\mathrm{const}.$$

*F_L_* (ε, n) [polarity function of Lippert’s equation]:

(3) $$F_{L\;}\left(\varepsilon ,\;n\right)=F\left(\varepsilon ,\;n\right)=\left[\frac{\varepsilon -1}{2\varepsilon +1}-\frac{n^{2}-1}{2n^{2}+1}\right]$$

Specific interaction is produced by neighboring molecules and are determined by specific chemical properties of both the fluorophore and solvent. In the present study different solvents decreased the intensity of emission and in only a few of the compounds was the spectrum red shifted. However, if the interaction only occurred in the excited state, the polar solvent had no effects on the absorption spectra, but if the interaction occurs in the ground state, then some change in the absorption spectrum is expected. The results showed that the solvents affected the intensity of absorption depending on the molecular structures of **I**–**V** [71], suggesting that possible interactions in the ground-state occur. If the fluorophore and the solvent are associated already in the ground state, then one would expect an immediate spectral shift upon excitation. If the fluorophore and solvent only associate in the excited sate, then the appearance of the specific solvent effect would depend on the rates of diffusion of the fluorophore and the solvent, which is like a quenching reaction. In general, among the isomers, the stronger solvent effect was observed on the compound **IV** (*meta* position of pyridine). 

In addition to specific solvent-fluorophore interactions, many fluorophores can form an internal charge-transfer (ICT) state or twisted internal charge-transfer (TICT). Unlike compounds **I** and **III**–**V**, no emission quenching was observed for compound **II** in any solvent, but the emission was affected by aprotic solvent polarity.

#### 2.2.5. Estimation of the Ground State Dipole Moment

Typically, the fluorophore has a larger dipole moment in the excited state (*μ_e_*) than in the ground state (*μ_g_*). Following excitation, the solvent dipoles can reorient or relax around *μ_e_*, which lowers the energy of the excited state. Due to the general description of solvent effects on the fluorophore, there is a continuous uniform dipole in a dielectric media. The model does not contain any chemical interactions, and hence cannot be used to explain the other interactions which affect the emission. These other interactions, such as hydrogen bonding or formation of charge transfer states, are sometimes detected as deviations from the general theory [7]. To evaluate the solvent effects on the fluorescence wavelength of the **I**–**V**, we considered the polarity parameters according to three Equations (2)–(4) [40,41,60,72].

The values obtained using calculations for the excited and ground state were evaluate the (ῡ_a_ − ῡ_f_), 1/2 (ῡ_a_ + ῡ_f_) are shown in Table S8. The ῡ_a_ and ῡ_f_ are the absorption and fluorescence maxima wave numbers (cm^−1^), ε and n are dielectric constant and refractive index of a solvent used Table S9. From the Equations (2)–(10), it follows that (ῡ_a_ − ῡ_f_) versus F(ε,n), (ῡ_a_ − ῡ_f_) versus, *F*_1_(ε,n) as well as 1/2 (ῡ_a_ + ῡ_f_) versus *F*_2_(ε,n), should be linear with slopes S, *S*_1_ and *S*_2_. The values of *μ_e_* − *μ_g_* and *μ_g_ μ_e_* were calculated according to Equations (7), (11) and (12):

(4) $$S=\;\frac{2{\left(\mu_{e}-\mu_{g}\right)}^{2}}{hca^{3}}$$

Bakhshiev’s Equation:

(5) $${\bar{\upsilon }}_{a}-{\bar{\upsilon }}_{f}=S_{1}F_{1}\;\left(\varepsilon ,\;n\right)+\mathrm{const}.\;$$

(6) $$F_{1\;\;}\left(\varepsilon ,\;n\right)=F_{1}\left(\varepsilon ,\;n\right)=\frac{2n^{2}+1}{n^{2}+2}\left[\frac{\varepsilon -1}{\varepsilon +2}-\frac{n^{2}-1}{n^{2}+2}\right]$$

(7) $$S_{1}=\;\frac{2{\left(\mu_{e}-\mu_{g}\right)}^{2}}{hca^{3}}$$

and Kawski–Chamma–Viallet’s–Bakhshiev’s equation:

(8) $$\frac{{\bar{\upsilon }}_{a}+{\bar{\upsilon }}_{f}}{2}=S_{2}F_{2}\;\left(\varepsilon ,\;n\right)+\mathrm{const}.$$

(9) $$F_{2}\left(\varepsilon ,\;n\right)=\frac{2n^{2}+1}{2\left(n^{2}+2\right)}\left[\frac{\varepsilon -1}{\varepsilon +2}-\frac{{\left(n\right)}^{2}-1}{{\left(n\right)}^{2}+2}\right]+\frac{3}{2}\left[\frac{n^{4}-1}{{\left(n^{2}+2\right)}^{2}}\right]$$

(10) $$S_{2}=\frac{2\left(\mu_{e}^{2}-\mu_{g}^{2}\right)}{hca^{3}}$$

The dipole moments of the *μ_g_* and *μ_e_* were estimated by the equations:

(11) μg= |S2−S12|[hca32S1]12

(12) μe= |S2+S12|[hca32S1]12

where *h* is Plank’s constant and *c* is velocity of light in vacuum and Onsager cavity radius (a), which was calculated using the report [70,73,74] as well as by Gaussian [64] according to their optimized geometry for **I**–**V (**Table 3). 

**Figure 9:**
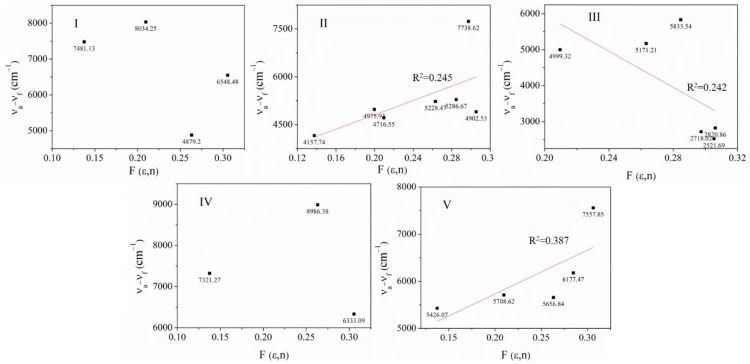
Plots of ῡ_a_–ῡ_f_ (cm^−1^) versus F(ε, n) of **I**–**V** in the solvents.

**Figure 10:**
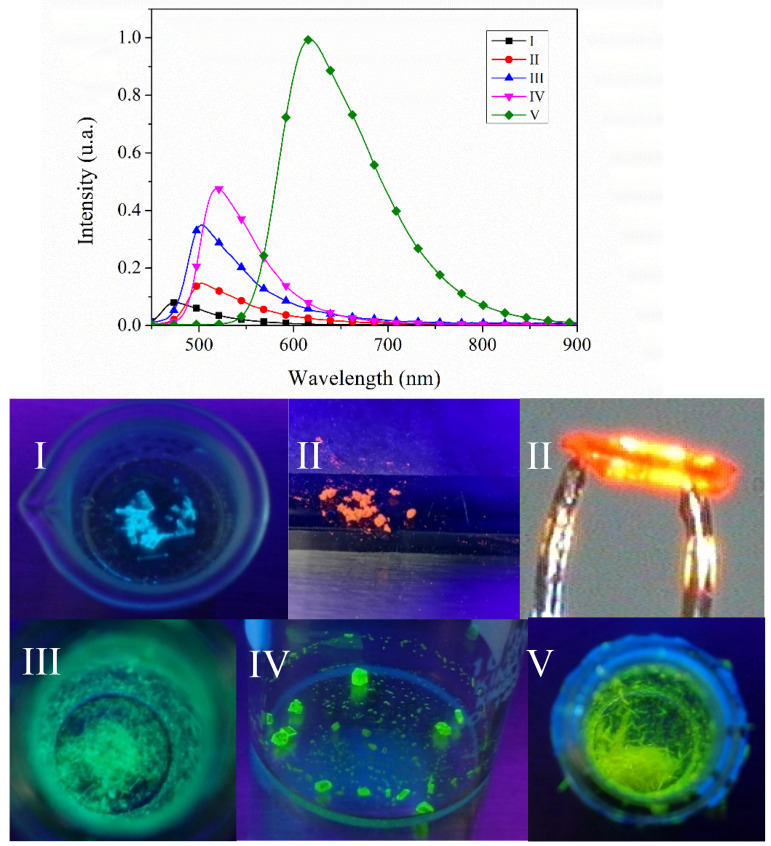
Emission spectra of compounds **I**–**V** and images of the crystals under UV lamp.

For the estimation of the dipole moments, we graphed (ῡ_a_ − ῡ_f_) versus F(ε,n), (ῡ_a_ − ῡ_f_) versus *F*_1_(ε,n) as well as 1/2 (ῡ_a_ + ῡ_f_) versus *F*_2_(ε,n), and did not observe a linear tendency for most of the solvents (Figure 9). Unlike several reports [40,41,60,71,72], which depend of the sensitivity of **I**–**V** to specific interactions with protic and aprotic and nonpolar solvents, these effects cannot prevent a quantitative interpretation of the emission spectra in terms of the orientation polarizability. In fact, the results show that the change of dipole moment is small (Table 3), and it is possible the interaction differs with a less polar solvent (CHCl_3_), in which all **I**–**IV** showed SCQ. However, the position of the nitrogen atom also plays an important role, because **IV** was the one that presented a SCQ in many solvents. possibly due to the disposition of the *meta* nitrogen atom to show greater interaction. The specific effects of solvents could reveal that the excited states of the dyes immediately start evolving into states in resonance [8,75] with the solvents. The HOMO and LUMO of the solvents used in the study are shown in Figure S15, even though the solvent dipole moment its well-known, in the excited state, it possibly will be different. Also, other intermolecular interactions such as internal charge-transfer (ICT) state or twisted internal charge-transfer (TICT) could occur. The study revealed **I** and **IV** exhibited more SCQ than **V** and **III**, but in the solid state the greater intensity was observed with **IV** > **V** > **III** >**I** (Figure 10). On the other hand, **II** did not exhibit quenching because of a TICT state, as compared to the molecular structure of **I** (Figure S11). Besides, internal charge-transfer (ICT) state or twisted internal charge-transfer (TICT) effects on the fluorescence are reduced in the solid-state phase of the compounds **I**–**V (**Figure 10). This is an indication of the different charge distribution in the excited state (S_1_) compared to the ground state (S_0_), as well as the non-covalent intermolecular interaction in **I**–**V**. 

All compounds were fluorescent in the solid-state (Figure 10). The quantum yields (Φ) (Table 2) were ranked **V** > **IV** > **III** > **II** > **I**. The fluorescence peaks in the solid state of **I** showed a blue shift of 46 nm whereas **II** displayed a red shift of 100 nm regarding **III**, **IV** and **V** (Table S10). A red shift of the emission spectrum in the solid state is common for most luminescent organic molecules. Therefore, the emission in solution versus the solid state could reflect a competition between the solute-solvent and solute-solute interactions to form the nuclei, and between the specific aggregation forces and packing in order to minimize repulsive interactions, which determine the structure of the new crystalline phase. 

### 2.3. Crystallographic Data

#### 2.3.1. Single Crystal X-ray Diffraction (SCXRD)

**Figure 11:**
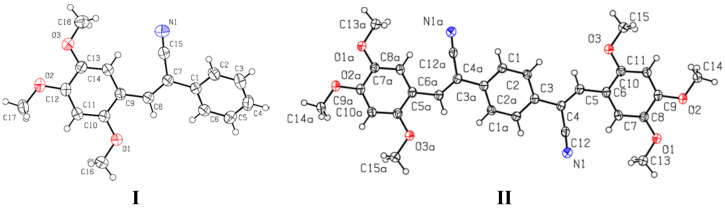
A view of the structures **I** and **II**, showing the atom-labelling scheme. Displacement ellipsoids are drawn at the 50% probability level.

Crystallographic data for **I** and **II** structure refinement parameters are summarized in Table S11 and for **III**–**V** in Table S12. The data for compounds **II**, **III** and **V** were collected at 110(2) K, whereas the data for compounds **I** and **IV** data were collected at room temperature. The ORTEP diagrams of the compound **I**–**II** are shown in Figure 11. 

Compound **I** was crystallized under different conditions and crystals with different appearance were obtained (**Ia** and **Ib**, Table S13). The crystal **I (Ia** and **Ib)** belongs to the monoclinic system with *P*2_1_/n with Z = 4 and the **II** crystal also belongs to the monoclinic system with space group of *P*2_1_/c and with Z = 2. 

**Figure 12:**
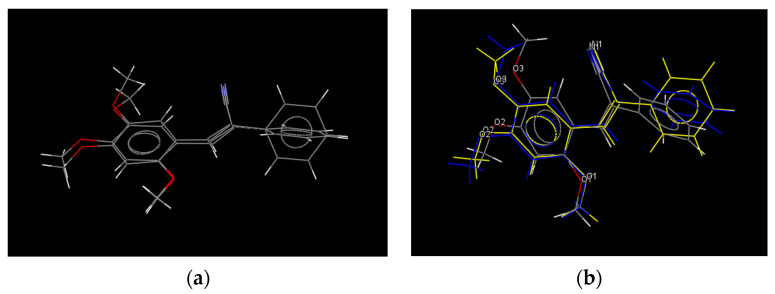
Overlay of the molecule structures of crystal **Ia, Ib** and **Ic**. (**a**) Overlay **Ia** and **Ic** and (**b**) overlary of **Ia**, **Ib** and **Ic**. [76].

Crystals of **Ia** and **Ib** were obtained in solvent **1** (DMSO) and **6** (EtOAc) and also in DMF (**I**c). The results from SCXRD showed that **Ia** and **Ib** were not polymorphic structures, but they exhibited differences in fluorescence emission of their crystals; this phenomenon had already been observed [51,52,53]. The Mercury software [76] showed that the molecular structures obtained in EtOAc (**6**) compared with DMSO (**1**) are conformers, but the crystals obtained from DMSO and DMF were not, implying a solvent polarity effect. In Figure 12, the molecular structures **Ia**, **Ib** and **Ic** are superimposed. A comparison of the selected bond lengths and torsion angles for **Ia**–**Ic** (Table 4) showed that these values of **Ib** ≈ **Ic** are similar, but not with **Ia**. The **I** molecular structures were non-planar, with torsion angles: −32.6(2)°, 30.5(2)°and 32.7(2)° and −30.7(2)°, 30.5(2)° and 30.5(2)°. The conformers formation in different solvents probably occurs due to the rotation and flexibility of the C(1)-C(7) bond between C(7) of the double bond CH=CCN- and C(1) of the aromatic ring, which is crucial for engineering and predicting crystal packing and, hence, their properties [77]. This idea was confirmed with the molecular structure of **II** (2*Z*, 2′*Z*)-2,2′-(1,4-phenylene)bis(3-(2,4,5-trimethoxyphenyl)acrylonitrile), in which the 2,4,5-(TM-phenyl) moiety is attached to -CH=CCN-, resulting in a molecular structure with high symmetry. The phenyl moieties attached to the double bound are constrained to adopt an almost planar geometry. The compound was synthetized from 1,4-diphenylacetonitrile with 2,4,5-(TMB) (Figure 11). When comparing the bond lengths found in the structures of **I** and **II**, no marked differences were observed for the double bonds and bonds between aromatic rings, see Table 4, but the bond length value of -C(4)-C(5) was 1.352(3) Å for **II.** Also, the torsion angles for the atoms C(2)-C(3)-C(4)-C(5) were −5.3(3)°, between C(1)-C(3)-C(4)-C(12) were −3.7(2)°, and between C(2)-C(3)-C(4)-C(12) were 176.47(15)° (Table 4). These values show that the molecular structure acquires greater planarity compared to **I**, which means that the electron density is more delocalized throughout the structure, affecting the intermolecular interaction and its molecular arrangement in the crystal packing.

**Figure 13:**
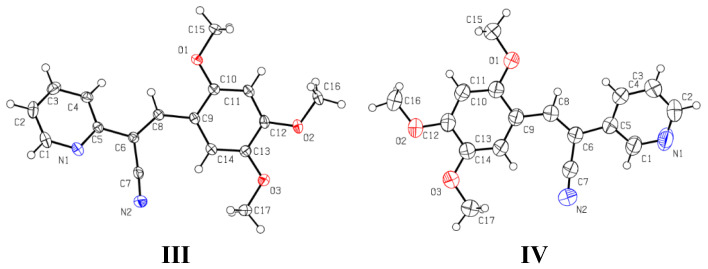

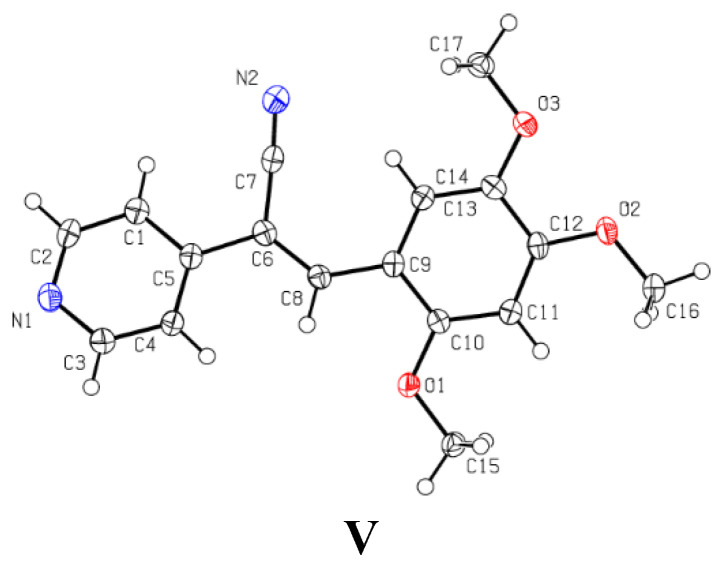
Displacement ellipsoid plots (50% probability level) of **III**–**V.**

The crystal structure of **III** with the *ortho* substituent refined into the orthorhombic space group *Pbca* with *Z* = 1. The crystal structure of **IV** refined into the triclinic space group *P*-1 with *Z* = 2, while that of **V** refined into the monoclinic space group *P*2_1_ with *Z* = 2 (Table S12). The ORTEP diagrams of the compounds **III**–**V** are shown in Figure 13. With respect to the molecular structures, the value of the length C=C bond in the three positional isomers was longer than the reported length of a conjugated double bond [78] (Table 5). This elongation is attributed to the electron withdrawing group (EWG)-CN, as well as the pyridine ring (EWG), when compared with **I**. The values of the C-C bond lengths found between the double bond and the aromatic rings are elongated as compared with a Csp^2^-Car [78] (Table 5). In contrast, the bond lengths C(8)-C(9) for the compounds **III**–**V** were shorter, 1.4481(17), 1.443(2), and 1.446(3), respectively, indicating that the MeO- substituents at 2,4,5- acted as electron donating groups (**EDG**) due to a resonance effect. Only the **III** and **V** molecular structures contain a quinoid structure in the aromatic ring of 2,4,5-three MeO-, which could affect the intermolecular interactions. 

The structures of **III**–**V** with the aromatic rings and the MeO- substituents are nearly planar [76]. However, the moieties composed of the pyridine rings and -CH = CCN- were found to be out of the molecular plane. In **III** the torsion angles between atoms C(4)-C(5)-C(6)-C(8), N(1)-C(5)-C(6)-C(7), and C(4)-C(5)-C(6)-C(7) have values of 29.39(18)°, 27.07(15)°, and −153.52(12)°, respectively. In compound **IV**, the atoms C(4)-C(5)-C(6)-C(8), C(1)-C(5)-C(6)-C(7), and C(4)-C(5)-C(6)-C(7) had values of 28.89°, 28.98° and −150.33°, respectively. For **V** the -CH = CCN- group and the aromatic ring were more coplanar with the values for the following torsion angles C(4)-C(5)-C(6)-C(8), C(1)-C(5)-C(6)-C(7), and C(4)-C(5)-C(6)-C(7) being 13.2(4), 11.8(3) and −167.2(2)°, respectively. The selected torsion angles for the **III**–**V** structures are listed in Table 6. These observations suggest that the intermolecular interactions play an important role in the relationship between molecular structure and crystal packing, which is typically found for compounds with the functional group -CH=CN-. This assertion was further corroborated with the molecular structures of compound **I**, which shows that the choice of solvents (DMSO, DMF, and ethyl acetate in this study) may induce small changes in molecular packing. 

#### 2.3.2. Full Molecular Interactions Maps

**Figure 14:**
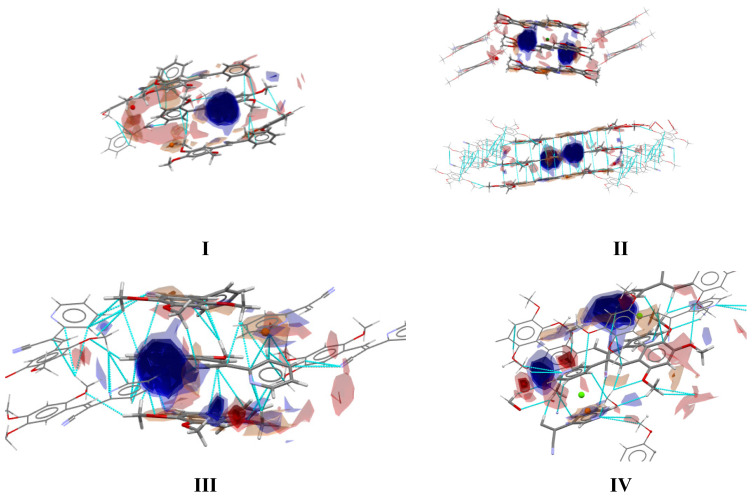

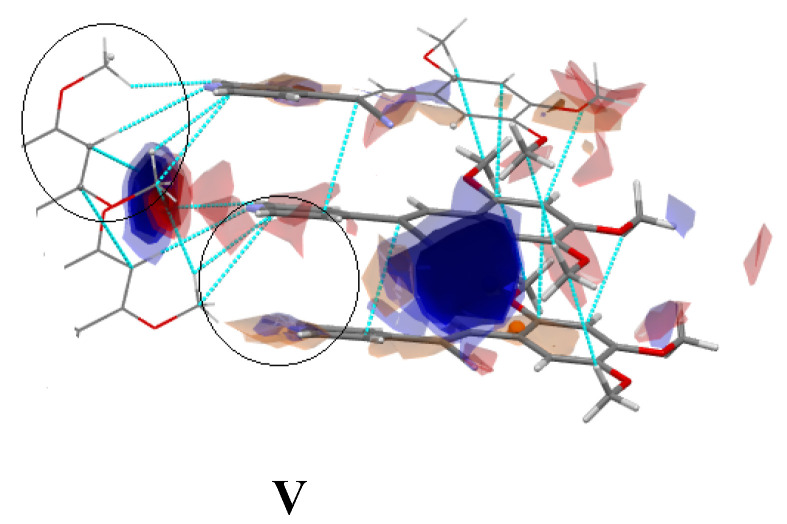
Interaction maps shown within packing patterns in the crystal structures of forms **I**–**V**. Regions of acceptor likelihood are shown in red, donors in blue and hydrophobic groups in brown.

The specific crystalline form of a compound has significant impact on its solid-state properties. A thorough understanding of molecular crystals can only be attained by considering and understanding the interplay of the full range of intermolecular interactions (and associated energies) that sustain molecules in their crystal lattices. The interaction maps were generated using the SuperStar methodology [56,79,80,81,82] using the program Mercury, the molecules of interest were split into IsoStar [76,79,80,81,82] central groups. For the compounds **I** and **III**–**V,** the potentially scatterplot is -CH_3_, pyridine, aromatic methoxy and -CN. A visualization of molecular interactions maps within the context of a crystal’s structure is shown in Figure 14. The generated interaction maps clearly showed 3 regions of very well-defined hotspots (the large brown, opaque and blue regions). As well as some more diffuse regions interactions (more transparent red, blue, and brown regions). According to the colors of the regions of the map, the structures did not denote a probability of locating a hydrogen bond. Blue regions denote acceptors atoms, and brown regions indicate hydrophobic preferences, the two slightly lower probability regions near the cyano groups. The positional isomers **III**–**V** show similar regions. There is also a small brown region indicating the possibility for a hydrophobic or π-π interaction. Looking at the region around the cyano groups, there are some short contacts available for interactions, but these are not directed toward the high-probability areas. From the acceptor probe maps (red contours), we can see that the main region of acceptor preference is satisfied by the cyano group from one molecule. Finally, there is a weak π-π interaction between the phenyl rings **I** and **II** that matches with one of the hydrophobic regions, and between the two pyridyl moieties in **III** [83].

#### 2.3.3. Molecular Packing of Compounds **I**–**V**

The analysis of the molecular packing mode provides important information about the charge transport between adjacent molecules which affects their optical properties such as absorption and emission. Molecular packing motif in **I** crystals is herringbone packing without π-π overlap (face-to-face) between adjacent molecules, Figure S18. Short contacts or intermolecular interactions are responsible for the different torsion angles present in each molecular packing of the crystals (Table 4). The **II** molecular packing shows a herringbone packing with a face-to- face slipped stacking π-π overlap and edge-to-edge between adjacent molecules (Figure S19). Furthermore, there is a 2D lamellar arrangement, being a more efficient molecular packing for exhibiting a high CT [84,85]. The molecular arrangements in **III**–**V** show that the pyridine, CN, and MeO- rings affect intermolecular interactions, causing a different molecular packing for these three compounds (see Figures S20–S22). Compound **III** shows a herringbone packing with edge-to-edge interactions without π-π (face-to-face) overlap between molecules; a zigzag arrangement is observed in the axis direction a. In the packing, there are short contacts between C(π)^_^H**^…^**C(π), Csp^3^-H**^…^**O, C(π)**^…^**Csp^3^-H, -CN and MeO-, but no π-π interaction to indicate possible charge transfer. The molecular arrangement of **IV** shows an arrangement in layers formed by dimers of molecules, presenting a lamellar structure in 2D. The number of short contacts is few, but a π-π interaction is observed (Figure S21). The contacts observed are O**^…^**HA, HB**^…^**O of MeO- groups. Between both sheets, the HA**^…^**N interaction forms face-to-face packing between pyridine-pyridine rings and the centroid distance is 3.682 Å, which allows greater charge transfer between the pyridine rings. Compound **V** shows a herringbone packing in the direction of c exe with sliding π-π overlap between adjacent molecules, with the corresponding centroid to centroid distance being 3.920 Å and the displacement distance is 1.834 Å, which it is an indication of a weak π-π interaction, based on the typical value reported for distances in an aromatic π-π interaction (>3.65 Å and offsets of 1.6–1.8 Å) (Figure S22) [76,84]. 

### 2.4. Quantitative Analysis of the Intermolecular Interactions of Compounds **I**–**V**

The PIXEL method is an extremely useful tool to explore the nature of supramolecular interactions that crystal engineers regularly employ to design molecular crystals [83,84,85,86,87,88]. This method can also identify intermolecular interactions that are perceived as binding, but are in fact associated with repulsion (“antagonist synthons”), or interactions that are characterized by insignificant attractive or repulsive forces (“neutral synthons”). According to the molecular structure and the CSD-materials [76] the **I**–**V** molecules did not show the generation of possible hydrogen-bonding networks with a knowledge-based assessment of the likelihood of each possible network. The lattice energies of the compounds calculated by the PIXEL program are shown in Table 7. The **II** crystal package has a higher lattice energy (−54.30 kcal/mol), indicating a higher stability for **II** and high intermolecular interaction. Among the three isomers **III**–**V** the lattice energy value differs slightly, being ranked as **III** > **V** > **III**. In all compounds, the total lattice energy value contributes to the dispersion energy (42–47%), the repulsion (28–33%), and the Coulombic energy (16–17%) while the contribution of E_po_l is very low. The dispersion energy contribution is interesting due to the behavior of the **I**–**V** in solution with different solvents. Possibly, competitive dispersion interactions exist with the solvent. Because large molecules make more solvent contacts than small molecules, they displace more solvent when they form a complex. In solution, the surfaces of all molecules are fully coated by other molecules and the change in dispersion energy for the interaction of two molecules in solution is small [89]. In an example, Hunter [89] displays a molecule of carbon tetrachloride solvent. Although there are no hydrogen-bond donors by molecular electrostatic potential surfaces plotted on the Van der Waals’ surface, the surface is quite strongly positive, due to the strongly electron withdrawing nature of the CCl_3_ group. This condition gives the chlorine atoms of carbon tetrachloride electrostatic properties equivalent to a weak hydrogen-bond donor [89]. The relevant parameter for comparing dispersion interactions in solution is therefore the interaction energy per unit surface area of contact. Our study of these new compounds in solution showed that the molecules interacted with solvents that contain oxygen and were independent of the moment polarity. 

**Figure 15:**
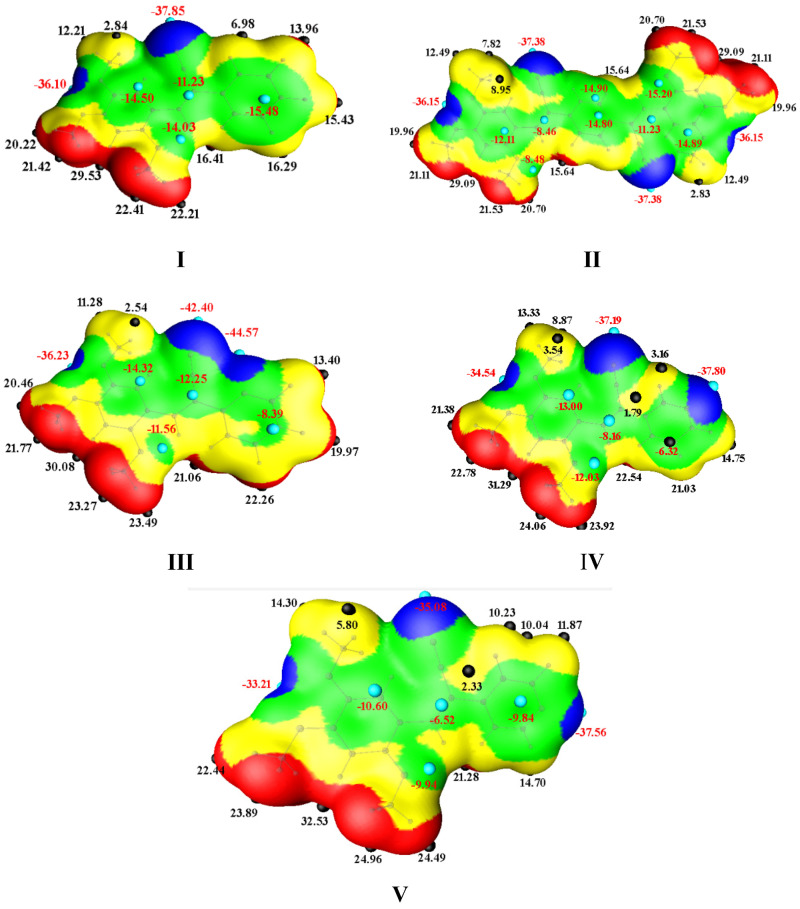
MEPS values of **I**–**V** are mapped over the electron density isosurface at 0.001 au. The positive (Vs, max) and negative (Vs, min) potentials are shown as small red and blue spheres, respectively.

In the present study, the molecular electrostatic potential surfaces, the maximum in the electrostatic potential on the van der Waals surface, of the **I**–**V** molecule were calculated. The area of the π-electron density and the MEPS could be used to examine a given property within a chemical series and propose a compound with improved features, or to investigate the interpretative abilities of some potential-related parameter for describing a certain aspect of the intermolecular interactions involved [83]. MEPS structures visualize local maxima and minima in charge distributions on the van der Waals surface, which represent donor and acceptor sites, respectively. From the MEPS plot for molecules **I**–**V** (Figure 15), the MEPS values for **I** are similar to those of the **II** molecular structure, but the MEPS values of **II** at the central part of the molecule are negative. The negative values suggest that intermolecular interactions occur and these central atoms are involved, as evident from the PIXEL analysis [90]. Compounds **III**–**V** display more positive MEPS in the range V_s,max_ = 30 to 32.5 kcal mol^−1^, due to the EWG effect of the pyridine group. The negative electrostatic potentials observed are localized on the nitrogen of the pyridine (−37 kcal/mol), CN (−42 to −35 kcal mol^−1^) and between the MeO-(**EDGs)**, which are in the *para* and *meta* positions on the phenyl ring. These results suggest that MeO- plays an important role in intermolecular interactions, that is, makes a greater contribution to the dispersion energy.

**Figure 16:**
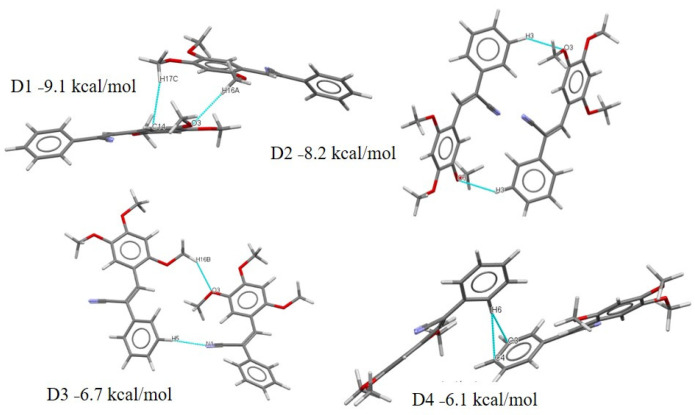
Different dimers (D1–D4) with interactions in the **I** crystal structure with respective interaction energies.

**Figure 17:**
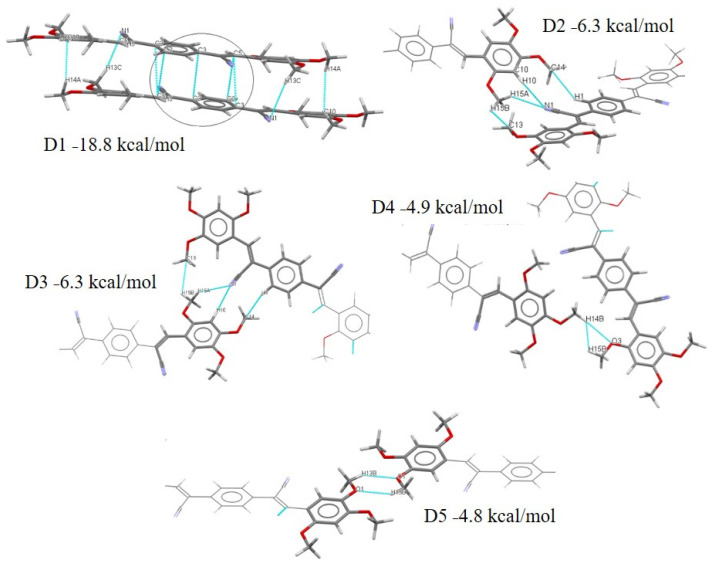
Different dimers (D1–D5) with interactions in the **II** crystal structure with respective interaction energies.

**Figure 18:**
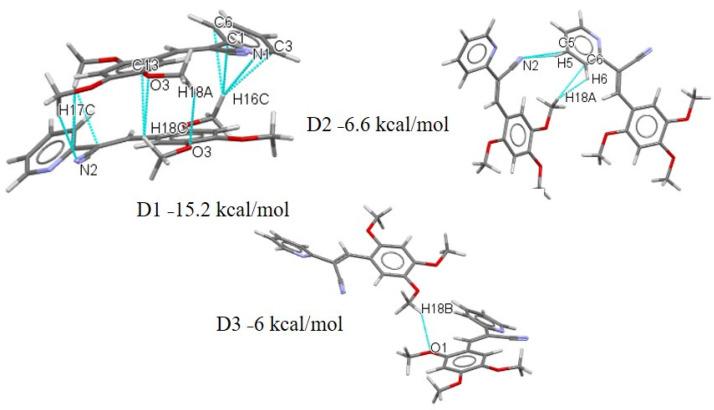
Different dimers (D1–D3) with interactions in the **III** crystal structure with respective interaction energies.

**Figure 19:**
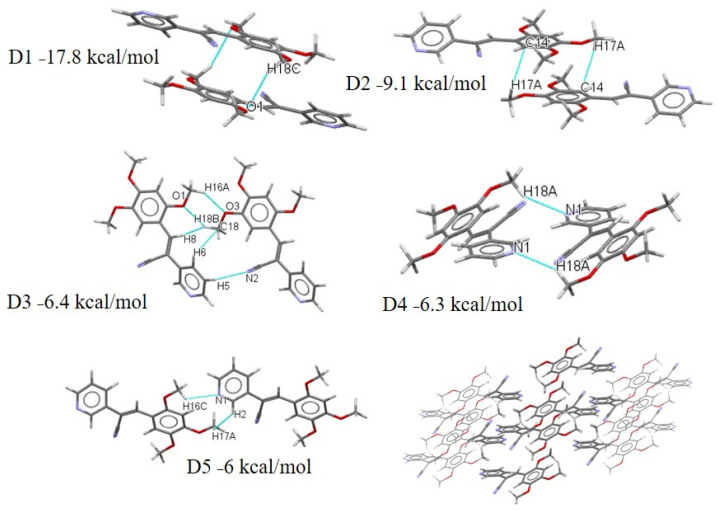
Different dimers (D1–D5) with interactions in the **IV** crystal structure with respective interaction energies.

**Figure 20:**
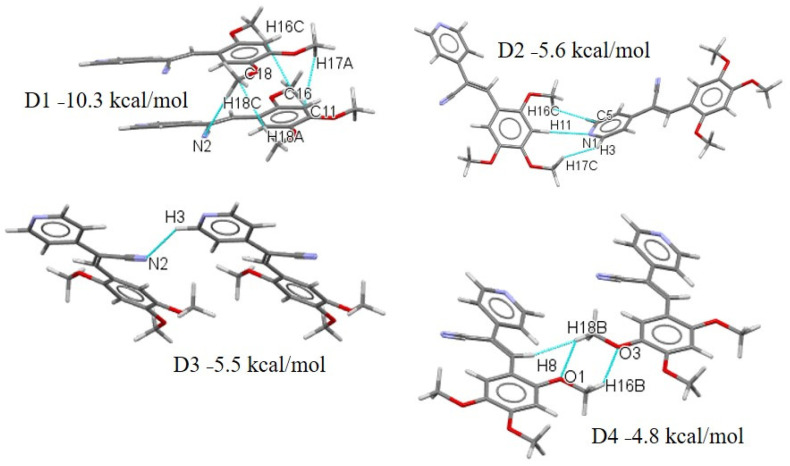
Different dimers (D1–D4) with interactions in the **V** crystal structure with respective interaction energies.

The dimer molecular pairs (motif) extracted from the crystal packing of compounds **I**–**V** are summarized in Table S17. The interactions associated with these energies for **I**–**V** are shown in Figure 16, Figure 17, Figure 18, Figure 19 and Figure 20. These values are very similar for reported compounds with acrylonitrile moieties with non-hydrogen-bonding interactions [43,44,46]. Also, most of the interaction energy values are within the expected range for these synthons calculated by PIXEL and can be used to identify intermolecular interactions that are perceived as binding [83,86,90,91]. The compound **I** exhibits lower energy compared with the **II** dimers, Table S17, and Figure 16, with the D1 distance for **I** between Cg1-Cg1 being larger than **II**. This result is an effect of intermolecular interaction through of π-π and charge density in **II**. The **II** crystals package shows a D1 stabilized by contacts C··· C. Interestingly, the carbon atom involved (Figure 17) in the interaction is from a double bond with a negative region (Figure 15 **II**), whereas the carbon atoms from the phenyl ring show a positive region. In the **III**–**V** isomers, the effect of the **EWG** pyridine group contributed to D1, with the dispersion energy being 48.16%, but the Coulombic energy being 8.36%. The D2, with an energy of −8.2 kcal/mol is due to the MeO- moieties in the *ortho* position. The D3 is stabilized by the one C-H···N form of the CN interaction and MeO- in the *ortho* with *para* position. The crystal package of compound **III** is stabilized by weak interactions C-H···N; C-H, C-H···O ·that contributed to the dimer D1 energy (−15.2 kcal mol^−1^). The D2 exhibits very modest binding energies involved the **EDG**-CN group with the H- of the MeO- in the *para* position, which perhaps interacts due to the negative charge closer to the oxygen atom and the protons of the MeO- substituent. Two synthons C-H···N and C-H···C6 have an energy value of −6.6 kcal mol^−1^ (Figure 18), but D3 exhibits only −6 kcalmol^−1^ energy, which is the same value for the C-H···O of the MeO- in the *meta* position. For **IV**, most of the interactions involved the MeO- functional substituents. D1 is stabilized by the C-H···O interaction with a binding energy of −17.8 kcal mol^−1^ (Figure 19). The interaction is from the oxygen of the MeO- in the *ortho* position which from MEPS (Figure 14) shows a more positive region and has a greater propensity to interaction to C-H of the MeO- in the *meta* position, which also is a positive region. These results explain the fact the dispersion energy contributes 48.32% to the total energy value of −17.8 kcal/mol. The D2 shows the C-H···C interaction with an energy of −9.1 kcalmol^−1^ with a higher percentage of energy dispersion (53.27%), as well as D3, D4 and D5 which have the same stabilization energy (−6.4 to −6.0 kcal/mol) (Figure 19). The dispersion energy together with the Coulombic contributions (26–24%), offset the repulsion contributions (Table S16). Clearly, the role of the MeO- substituents is important. The molecular shells calculation by Mercury starting form a central molecule to the neighboring molecules possibly indicates π-π stacking between Py-Py (Figure S16). The D5 is interesting because the interaction between C-H···N and C-H···H, i.e., C-H from the MeO- in the *ortho* position and MeO- in the *para* position. The MEPS structures show the methyl groups have more positive charge. 

For compound **V** (Figure 20), the D1 stabilization energy is of −10.3 kcal mol^−1^. Interestingly, the large contribution of dispersion energy (52.94%), indicates interactions that are like the compound **III**, whose dimer structure is further stabilized by the one C-H···N from the CN interaction. The D2, the moiety composed by two MeO- in positions 2 and 4 (see the positive region, Figure 15, **V**), interacts with the pyridine ring, which is an **EWG**. The D3 confirms that the interaction of C-H···N involved the -CN and D4 the dispersion contribution was due to the MeO- moieties. However, they were slip-packed by weak *π*-stacking interactions, which may be affecting the charge properties of the crystals, contrary to the crystals of **IV**. The other interactions are C-H··· N of -CN and C-H··· C (Figure 20). 

### 2.5. Natural Bond Orbital (NBO) Analysis

The NBO **I**–**V** were performed using the NBO program carried out through the GAUSSIAN software [64] at the DFT/B3LYP level. The natural bond orbital analysis is a method for studying intra- and intermolecular bonding and interactions among bonds, besides providing a convenient basis for investigating charge transfer or conjugative relations in molecular structure [92,93,94,95,96]. The greater stabilization energy E(2) value indicates more intensive interaction amongst the electron acceptors and donors, i.e., the higher electron donating ability and superior degree of conjugation of the whole system. For the pyridylacrylonitrile, 2,4,5-TMphenyl were found several interactions including lone pairs of electrons (n) on the oxygen and nitrogen atoms (Tables S14–S16 and S18). The NBO comparison of molecule **I** displays a lower stabilization energy, which could explain why the molecule does not have a propensity to participate in charge transfer stabilization. Similarly, molecule **II** displays strong interactions from π donor to -π* acceptor which are concentrated at the phenyl group. The selected second order perturbation values (Table S18) indicate that more than one BD donor interacted with the same BD acceptor, which means that the stabilization energy in the crystal should support the role of the charge transfer on the optical properties in the studies with compounds **I**–**V**. Within the pyridylacrylonitrile moiety for the **III**–**V** compounds, the larger energy (stabilization energy) E(2) value occurs with four strong intermolecular hyper-conjugative interactions of π electrons of pyridine bonds to π* that involved the -C=C-CN, with an energy range of 39–20 kcal/mol. Additionally, within the 2,4,5-TMP-phenyl group we also observed larger E(2) values which correlate with a donor type π to an acceptor π*, with values of 32–27 kcal/mol (Table S18). Besides NBO analysis gave for **III**-**IV** the intermolecular interactions with a stabilization energy (n→π* and n→σ*) from oxygen and nitrogen to bonds of the phenyl rings with stabilization energies in the range of 41–14 kJ/mol obtained from the lone electron pair of the nitrogen of the pyridine to σ*s of the bonds of the pyridine ring. These calculations are an indication of the strong interaction of the dimers in the crystal package, and of possible charge transfer. The intramolecular charge transfer (ICT) formed by electron delocalization from σ→σ* and n→σ* causes stabilization of the system. 

## 3. Materials and Methods

### 3.1. Materials and Instrumentation

2,4,5-Trimethoxybenzaldehyde (2,4,5-(TMB)), 2-pyridylacetonitrile (2-PyAcN), 3-pyridylacetonitrile (3-PyAcN), 4-pyridine in acetonitrile hydrochloride, (HCl_4_-PyAcN) and 1,4-phenylenediacetonitrile **(**1,4-PhDAcN) were acquired from Aldrich Chemical Co. (Milwaukee, WI, USA). Phenylacetonitrile (PhAcN) and potassium hydroxide (KOH) were acquired from Alfa Aesar (Tewksbury, MA, USA) and J.T. Baker (Phillipsburg, NJ, USA), respectively. All chemical reagents were used without purification. Melting points were measured with an SEV (0–300 °C) apparatus (SEV, Puebla, México) and were reported as uncorrected values. IR spectra of the products were recorded on a Vertex 750 FT-IR spectrophotometer (model 70, Bruker Optics, Ettlingen, Germany) by attenuated total reflectance (ATR). ^1^H-NMR and ^13^C NMR spectra were obtained in DMSO-*d*_6_ on a 500 MHz NMR spectrometer (Bruker, Guadalajara, México). The electron ionization (EI) spectra were acquired on a Jeol MStation 700-D mass spectrometer (Jeol USA, Peabody, MA, USA).

### 3.2. Absorbance and Emission (UV-Vis and PL)

The absorbance spectra were measured using a Cary 300 (Agilent, Mexico City, Mexico) spectrometer equipped with a deuterium and halogen lamp. Emission spectra (PL) were acquired with a QE-Pro-FL (Ocean Optics, Dunedin, FL, USA); a UV-lamp mineral light with emission at 350 nm was used as the excitation source. J-V curves were acquired with a Keithley 2450 source-meter (Tektronix, Beaverton, OR, USA).

### 3.3. Single Crystal X-ray Diffraction (SCXRD)

All reflection intensities for the compounds **II, III** and **V** were measured at 110(2) K using a SuperNova diffractometer (Agilent Technologies Yarnton, Oxfordshire, UK) equipped with an Atlas detector with Cu Kα radiation (λ = 1.54178 Å) under the program CrysAlisPro (Version CrysAlisPro 1.171.39.29c, Rigaku OD, 2017) [97]. The same program was used to refine the cell dimensions and for data reduction. The structure was solved with the program SHELXS-2018/3 [98] and was refined on *F*^2^ with SHELXL-2018/3 [98]. Analytical numeric absorption correction using a multifaceted crystal model was applied using CrysAlisPro [97]. The temperature of the data collection was controlled using the system Cryojet (manufactured by Oxford Instruments, Abingdon, Oxfordshire, UK). The H atoms were placed at calculated positions using the instructions AFIX 43 or AFIX 137 with isotropic displacement parameters having values 1.2 or 1.5 Ueq of the attached C atoms. The structures of **II, III** and **V** are ordered. For **V**, the absolute configuration has been established by anomalous-dispersion effects in diffraction measurements on the crystal, and the Flack and Hooft parameters refine to 0.09(11) and 0.12(9), respectively.

Suitable crystals for compounds **I** and **IV** were selected carefully using an optical microscope, and the X–ray intensity data was collected on a Xcalibur, Gemini diffractometer (Agilent Technologies Yarnton, Oxfordshire, UK). The crystal was kept at 293 K during data collection. The crystal structures were solved by direct method with the program SHELXS2014 [98] in Olex2 [99] platform and all the non-hydrogen atoms were refined anisotropically using the SHELXL2014 [98]. All the hydrogen atoms were placed in ideal geometry positions and constrained to ride on their parent atoms.

CCDC 2038534, 2038536, 2038535, 2045035, 2045040, 2045041, 2045043 contains the supplementary crystallographic data for this paper. These data can be obtained free of charge via http://www.ccdc.cam.ac.uk/conts/retrieving.html; accessed on August 2020 (or from the CCDC, 12 Union Road, Cambridge CB2 1EZ, UK; Fax: +44 1223 336033; E-mail: deposit@ccdc.cam.ac.uk)

### 3.4. PIXEL Energy and Quantum Chemical Calculations 

All the quantum chemical calculations were performed with the Gaussian 09 program package [64]. The crystal structure geometry of **I**–**V** was used as a starting geometry optimization calculation. The constraints free optimization was carried out by using M06-2X/cc-PVTZ [65,100], which the level of theory with Grimme’s D3 dispersion corrections also incorporate [101]. The vibrational frequency was calculated for the optimized geometry in a vacuum and solvent phase to ascertain the global minima on the potential energy surface and were found to have no negative frequencies. To explore the solvent influence, we used the conductor-like polarizable continuum model (CPCM) [102] for all the solution phase calculations. Time-dependent DFT (TDDFT) Kohn-Sham formalism [103] was used to calculate the absorption properties of the optimized geometries with M06-2X/cc-PVTZ level of theory. 

The interaction energies (Etot) and lattice energies was calculated using the PIXEL method (in the CLP computer program package, version 12.5.2014) [104]. The interaction energy (Etot) was calculated for various molecular pairs extracted from the respective crystal structure as related to the corresponding symmetry elements as described as described [57,104,105]. The C-H bond lengths were adjusted to neutron diffraction values (C-H = 1.089 Å) before the PIXEL calculations. For the PIXEL calculations, the electron density of the molecules was obtained at MP2/6-31G** level of theory using Gaussian09. Natural Bond Orbital (NBO) was calculated with M062x/cc-pVTZ level theory. The NBO analyses were applied for investigating donor–acceptor interactions in the compound [106]. The quantitative molecular electrostatic potentials for all compounds were computed and visualized on the 3D surface using the Multiwfn program [107] and Visual Molecular Dynamics program (VMD) [108]. The quantitative molecular electrostatic potentials were mapped on the electron density isosurface at 0.001 a.u. The two different orientations of the MEPS of the isolated molecule and the locations of various most positive and negative potentials along with their values, designated as V_s,max_ and V_s,min_, respectively, were determined. 

## 4. Conclusions

Compounds **I**–**V** were completely characterized and the study of the absorption properties in solvents of different polarity revealed behavior of hypochromic or hyperchromic phenomena. Compounds **I**–**V** were sensitive to solvent effects regardless of the dipole moment of the solvent. In general, a linear dependence was not observed as a function of polarity, but rather with solvents that are made up of O atoms, especially acetone and EtOAc. This result may indicate that molecules **I**, **III**–**V** preferentially engage in dispersion-type intermolecular interactions. It also reinforces the importance of intermolecular interactions. These observations reinforce the fact that the molecules of **II**, despite having a fundamental dipole moment of approximately zero, do not suffer the same solvent effects as compounds **I**, **III**–**V**. On the one hand, these results may reflect the effects of ICT, but our study also shows that intermolecular interactions between molecules of **II** are of higher energy. On the other hand, the effect of the solvent in solution through the Stokes Shift and the Lippert’s, Bakhshiev’s, and Kawski-Chamma-Viallet’s-Bakhshiev’s equations allowed us to evaluate a non-linear dependence as a function of the solvent, which indicated that the emission in the solid state may be due to intermolecular interactions. These studies were corroborated with PIXEL, MEPS, and NBO data which gave information that extended our understanding of the effect in the solid state. Our observations are important because crystallization is a process that occurs when a set of molecules comes together to form a condensed array with regularly repeating interactions. A fundamental understanding of these interactions is therefore crucial in the analysis, evaluation, and prediction of crystal forms. Finally, the emission of a compound depends on the intermolecular interactions and when these energies are weak, as for **I** (emission towards the blue, 472 nm), **III** ≈ **IV** (501–502 nm), **V** (518 nm) and **II** (616). Measurement of the quantum yields, which were ranked **V** ≈ **IV** > **III** > **II** >> **I**, corroborated the formation of excimers or TICT, which affect the efficiency of the material. However, for compounds derived from isomeric acrylonitriles with pyridine, the *meta* position was the most efficient in terms of quantum yield. Thus, the π-π interactions due to the pyridine ring contribute without the formation of excimers as is the case of the **II** compound (red emission is an indication of high negative energy and blue emission indicates positive energy interaction).

## Figures and Tables

**Table 1:** Absorption wavelength (nm), excitation energy (eV) and oscillator strengths (*f*) of **I**–**V** compounds. Experimental and calculated at theory level of M062x/cc-pVTZ.

I					II				
λ (nm)	E (eV)	*f*	Major Contribution (%)	λ_exp_ (nm)	λ (nm)	E (eV)	*f*	Major Contribution (%)	λ_exp_ (nm)
Gas									
345	3.58	0.705	HOMO→LUMO(97)	376	380	3.25	1.713	H − 1→L + 1(12) HOMO→LUMO(86)	416
276	4.49	0.165	H − 1→LUMO(86)	299	288	4.29	0.116	H − 3→L + 1(10) H − 2→LUMO(60) H − 1→L + 1(19)	281
246	5.03	0.006	H − 2→LUMO(40) H − 1→L + 1(12) HOMO→L + 1 (30)	-	252	4.90	0.118	H − 2→LUMO(13) H − 1→L + 1(58)	254
237	5.21	0.074	H − 3→LUMO(20) H − 1→LUMO(12) HOMO→L + 2(33) HOMO→L + 3(27)	241	229	5.40	0.152	H − 4→LUMO(55) HOMO→L + 2(37)	-
224	5.53	0.016	H − 3→LUMO(47) H − 2→LUMO(14) HOMO→L + 2(23)	-	216	5.72	0.185	HOMO→L + 3(19) HOMO→L + 5(46)	-
			**III**				**IV**		
347	3.57	0.691	HOMO→LUMO(97)	393	348	3.55	0.673	HOMO→LUMO(97)	387
275	4.49	0.204	H − 1→LUMO(85)	276	277	4.46	0.168	H − 1→LUMO (85)	306
260	4.75	0.001	H − 3→LUMO(39) H − 3→L + 1(40)	250	261	4.74	0.012	H − 3→LUMO (39) H − 3→L + 1 (40)	349
248	4.98	0.039	H − 4→LUMO(10) H − 2→L + 1(12) H − 1→L + 1(51)	243	247	5.01	0.028	H − 3→LUMO(10) H − 1→L + 1 (12) HOMO→L + 1(51)	
237	5.21	0.001	H − 3→LUMO(14) H − 3→L + 1(43) H − 3→L + 2(12)		239	5.18	0.014	H − 3→LUMO(14) H − 3→L + 1 (43) H − 3→L + 2 (12) H − 2→LUMO (10)	
			**V**						
352	3.51	0.661	HOMO→LUMO(97)	393					
278	4.45	0.193	H − 1→LUMO(88)	310					
266	4.64	0.005	H − 3→LUMO(60) H − 3→L + 2(25)	248					
244	5.06	0.014	H − 2→LUMO(66) HOMO→L + 1(12)	-					
235	5.25	0.071	HOMO→L + 2(37) HOMO→L + 3(35)	-					

**Table 2:** Stokes Shift (cm^−1^) in solution, solid state, and quantum yield in solid state of **I**–**V**.

	Stokes Shift (cm^−1^)				
Solvent	I	II	III	IV	V
DMSO	4879.2	5228.47	5171.21	8986.38	5656.84
MeOH	--	14,842.76	2820.86	--	7557.85
EtOAc	--	4902.53	2521.69	--	6177.47
AcCN	8034.25	5286.67	5833.54	--	5708.62
Acetone	7481.13	4716.55	4999.32	7321.27	5426.07
CHCl_3_	6548.48	4975.93	--	6333.09	--
THF	--	4157.74	--	--	--
MeOH/acetone	--	7738.62	2718.02	--	--
*η*	2.75	2.50	2.73	2.73	2.70
powder	1644.61	322.52	1638.19	1615.48	571.5
Φ* = (%)	14.58	40.13	80.72	138.16	148.72

Φ* = Quantum yield, *η* = hardness value, see Equation (1).

**Table 3:** Onsager Cavity Radii, a, (in Å), *μ_e_*, *μ_g_* dipole moments (D) of **I**–**V.**

	*μ_e_* − *μ_g_*	*μ_g_* D	*μ_e_*	Φ°	a *	a **	*μ_g_* (D)	*μ_e_*
**I**	-	-	-		4.95	5.32	5.71	7.63
**II**	1.52	7.58	13.1	33.04	5.93	6.23	0.0003	12.69
**III**					4.92	5.55	7.22	8.13
**IV**	2.13	3.72	5.65	49.89	4.92	5.49	7.69	8.09
**V**	1.31	9.2	3.97	38.02	4.92	5.21	7.15	7.45

a = Onsager cavity radius; * = calculated according to [70], ** = calculated according to [64].

**Table 4:** Selected bond lengths (Å) and torsion angles (°) for the molecule structures of **I** obtained at different crystallization conditions and of **II**.

Assigned [77]	Bond Length	Ia	Ib	Ic	II	
Double bond C*sp2*=C*sp2* (overall) [77] 1.316 If it is conjugated with Ar. 1.339	C(7)-C(8)	1.3483(18)	1.3457(18)	1.3492(16)	C(4)-C(5)	1.352(3)
Bond C*ar*≈C*ar* C≈C (overall) 1.380 1.384	C(9)-C(10) C(9)-C(14) C(10)-C(11) C(13)-C(14)	1.4058(18) 1.4082(18) 1.3994(18) 1.3762(18)	1.4073(18) 1.4078(18) 1.3961(18) 1.3759(18)	1.4043(16) 1.4067(16) 1.3965(15) 1.3742(16)	C(6)-C(11) C(6)-C(7) C(10)-C(11) C(7)-C(8)	1.403(3) 1.408(3) 1.404(2) 1.387(2)
C*sp2*-C*ar* (overall) 1.483 (conjugated)1.470, 1.488	C(1)-C(7) C(8)-C(9)	1.4897(17) 1.4548(17)	1.4898(17) 1.4569(17)	1.4861(15) 1.4536(15)	C(3)-C(4) C(5)-C(6)	1.480(2) 1.455(2)
	**Ia**	**Ib**	**Ic**	**II**		
Atoms	(°)			Atoms		(°)
C(6)-C(1)-C(7)-C(8)	−32.6(2)	32.9(2)	32.7(2)	C(2)-C(3)-C(4)-C(5)		−5.3(3)
C(2)-C(1)-C(7)-C(15)	−30.7(2)	30.5(2)	30.5(2)	C(1)-C(3)-C(4)-C(12)		−3.7(2)
C(6)-C(1)-C(7)-C(15)	148.9(1)	−148.89(15)	−148.8(1)	C(2)-C(3)-C(4)-C(12)		176.47(15)
C(15)-C(7)-C(8)-C(9)	0.5(2)	−0.2(3)	−0.6(2)	C(12)-C(4)-C(5)-C(6)		−3.1(3)
C(1)-C(7)-C(8)-C(9)	−177.9(1)	177.85(14)	177.8(1)	C(3)-C(4)-C(5)-C(6)		178.77(17)
C(7)-C(8)-C(9)-C(10)	169.8(1)	−170.14(16)	−169.9(1)	C(4)-C(5)-C(6)-C(11)		175.14(18)
C(7)-C(8)-C(9)-C(14)	−7.9(2)	7.8(3	8.0(2)	C(4)-C(5)-C(6)-C(7)		−6.0(3)
C(14)-C(9)-C(10)-O(1)	−178.9(1)	178.8(1)	178.8(1)	C(7)-C(6)-C(11)-O(3)		175.56(15)
C(8)-C(9)-C(10)-O(1)	3.1(2)	−3.1(2)	−3.2(2)	C(5)-C(6)-C(11)-O(3)		−5.5(2)
C(10)-C(11)-C(12)-O(2)	178.8(1)	−178.8(1)	−178.8(1)	O(2)-C(9)-C(10)-C(11)		−177.78(17)

**Table 5:** Select bond lengths (Å) for the molecular structures of **III**–**V**.

Assigned [77]	III		IV	IV
Double bond C*sp2*=C*sp2* (overall)1.316 * with Ar. 1.339	C(6)-C(8)	1.3560(16)	1.351(2)	1.358(3)
Bond C*ar*≈C*ar* C≈C (overall) 1.380 1.384	C(9)-C(10) C(9)-C(14)C(10)-C(11) C(13)-C(14)	1.4060(16) 1.4110(17) 1.3952(17) 1.3747(17)	1.405(2) 1.407(2) 1.398(2) 1.377(2)	1.405(3) 1.404(4) 1.402(3) 1.376(3)
C*sp2*-C*ar* (overall) 1.483 (conjugated) 1.470, 1.488	C(5)-C(6) C(8)-C(9)	1.4869(17) 1.4481(17)	1.491(2) 1.443(2)	1.486(3) 1.446(3)

* when conjugated.

**Table 6:** Selected torsion angles (°) for the molecular structures of **III**–**V**.

Atoms	III	IV	V
C(4)-C(5)-C(6)-C(8)	29.39(18)	28.89	13.2(4)
N(1)-C(5)-C(6)-C(7)	27.07(15)		
C(1)-C(5)-C(6)-C(7)		28.98	11.8(3)
C(4)-C(5)-C(6)-C(7)	−153.52(12)	−150.33	−167.2(2)
C(7)-C(6)-C(8)-C(9)	−1.7(2)	1.18	3.6(4)
C(5)-C(6)-C(8)-C(9)	175.10(12)	−177.97	−176.9(2)
C(6)-C(8)-C(9)-C(10)	−173.62(13)	179.17	−174.3(2)
C(6)-C(8)-C(9-C(14)	4.3(2)	0.88	7.1(4)
C(14)-C(9)-C(10)-O(1)	178.97(10)	178.23	−177.7(2)
C(8)-C(9)-C(10)-O(1)	−2.97(16)	−0.17	3.6(3)
C(10)-C(11)-C(12)-O(2)	−178.25(12)	178.11	−179.4(2)

**Table 7:** Lattice energies (kcal mol^−1^) partitioned into Coulombic, polarization, dispersion and repulsion contributions for all compounds.

Crystal	E_coul_	E_pol_	E_dis_	E_rep_	E_tot_
**Ia**	−14.31	−5.37	−39.07	23.70	−35.06
**Ib**	−14.55	−5.59	−39.60	24.71	−35.03
**Ic**	−14.38	−5.47	−39.34	24.23	−34.96
**II**	−25.50	−9.76	−71.91	52.86	−54.30
**III**	−19.01	−7.93	−44.43	33.24	−38.12
**IV**	−14.02	−5.28	−40.12	23.18	−36.25
**V**	−12.97	−7.24	−45.84	31.64	−37.45

## Data Availability

No data is reported.

## Supplementary Materials

The following are available online, 1.0: General procedure for the compounds (**I**–**V**). Table S1: Reaction conditions used for obtaining **I**–**V**. 2.0: Characterization IR, ^1^H-NMR and EI of the compounds (**I**–**V**). Figure S1: IR spectra of the compounds **I**, **II**, **III**, **IV** and **V**. Scheme S1: **III** is when X = N; Y = C; and Z = C: **IV** is when X = C; Y = N; and Z = C: **V** is when X = C; Y = C; and Z = N. Table S2: Chemical shift values (ppm) and coupling constant (J_Hz_) values of compound compounds **I**. Table S3: Chemical shift values(ppm) and coupling constant (Hz) values of compound compounds **II**. Table S4: chemical shift values(ppm) and coupling constant (Hz) values of compounds **III**–**V**. Figure S2: ^1^H-NMR spectra of the compounds **I**, **II**, **III**, **IV** and **V** in CDCl_3_. 3.0: Absorption spectroscopy in solution. Figure S4: Absorption spectra of compound **I** in different solvents. Figure S5: Absorption spectra of compound II in different solvents. Figure S6: Absorption spectra of compound **III** in different solvents. Figure S7: Absorption spectra of compound **IV** in different solvents. Figure S8: Absorption spectra of compound **V** in different solvents. Table S5: Photophysical properties **λ_ab_**_s1,_ **λ_ab_**_s2,_ **λ_ab_**_s3,_ (nm) and ε(M^−1^ cm^−1^) of the **I**–**V** compounds in different solvents. Figure S9: Absorption spectra of **I** calculated with at theoretical level of m062x/pVTZ in different solvents. Figure S10: Absorption spectra of **II**, calculated with at theoretical level of m062x/pVTZ in different solvents. Figure S11: Absorption spectra of **III**, calculated with at theoretical level of m062x/pVTZ in different solvents. Figure S12: Absorption spectra of **IV**, calculated with at theoretical level of m062x/pVTZ in different solvents. Figure S13: Absorption spectra of **V**, calculated with at theoretical level of m062x/pVTZ in different solvents.Table S6: Experimental and calculated at theory level of m062x/cc-pvtz for **I**–**V** compounds. Absorption wavelength (nm), excitation energy (eV) and oscillator strengths (*f*) in different solvents. Figure S14: Schematic diagrams of HOMO and LUMO comparison by DFT calculations of acrylonitrile’s compounds contain ring A substituted with F, Cl, Br, 2,4,5-TMO, -N(CH_3_)_2_, N(Ph)2, Cz and ring (B) is a phenyl ring or pyridine ring in position *ortho*, *meta* and *para*. Figure S15: Graph of HOMO and LUMO calculations, energy gap with theory level of m062x/pVTZ. Table S7: DFT calculations of DMSO, MeOH, AcCN, Acetone THF, EtOAc, Chloroform. Figure S16: Images of the emission **I**–**V** solution under UV lamp in different solvents at concentration of 0.001 mM. Figure S17: Dipole moment of **I**–**V** performed with a theory level at m062x/pVTZ. Table S8: Wave numbers for the absorption and fluorescence emission maxima of the **I**–**V** in different solvents and the Stokes shift. Table S9: Dielectric constant and refractive index of various solvents, as well as Lippert’s, Bakhshiev’s and Kawasaki-Chamma-Violet’s polarity parameters. Table S10: Wave numbers for the absorption and fluorescence emission maxima of the **I**–**V** in solid state and the Stokes shift. Table S11: Crystallographic data for the structures of **I** and **II**. Table S12: Crystallographic data for the structures of **III**–**V**. Table S13: Crystal morphology for **I**–**V**. Figure S18: herringbone packing or **I** slipped stacking without π-π overlap between adjacent molecules. Figure S19: Herringbone packing of **II** with slipped stacking (i) with very slip weak π–π overlap between adjacent molecules and (ii) a view down the bc crystallographic plane. Figure S20: Molecular packing of **III** slipped stacking without π-π overlap occurring between adjacent molecules. Figure S21: Molecular packing of **IV** which shows a structure of 2D supramolecular lamella and π-π overlap stacking occurring between Py-Py rings. Figure S22. Herringbone packing or **V** slipped stacking without π-π overlap occurring between adjacent molecules. Table S14. Second order perturbation theory analysis of Fock matrix in NBO basis for **I** and **II**. Table S15. Second order perturbation theory analysis of Fock matrix in NBO basis for **III** and **IV**. Table S16. Second order perturbation theory analysis of Fock matrix in NBO basis for **V**.

## Sample Availability

Samples of the compounds are not available from the authors.
